# Tumor-derived exosomes induce neutrophil infiltration and reprogramming to promote T-cell exhaustion in hepatocellular carcinoma

**DOI:** 10.7150/thno.104557

**Published:** 2025-02-03

**Authors:** Wenchao Bi, Xue Li, Yu Jiang, Tongtong Gao, Huajun Zhao, Qiuju Han, Jian Zhang

**Affiliations:** 1 Institute of Immunopharmaceutical Sciences, School of Pharmaceutical Sciences, Shandong University, Jinan, China.; 2 Department of Medicinal Chemistry, Key Laboratory of Chemical Biology (Ministry of Education), School of Pharmaceutical Sciences, Shandong University, Jinan, China.

**Keywords:** Hepatocellular carcinoma, Tumor-associated neutrophils, Exosomes, miR-362-5p, T-cell exhaustion.

## Abstract

**Rationale:** High neutrophil infiltration in hepatocellular carcinoma (HCC) is associated with a poor prognosis in patients with HCC. Tumor-derived exosomes (TDEs) have been proven to be important in the reprogramming of tumor-associated neutrophils (TANs), but the roles and mechanisms have not been fully clarified.

**Methods:** The roles of HCC-exosome-reprogrammed neutrophils on tumor progression were evaluated in the DEN/CCl_4_-induced HCC mouse model by blocking neutrophil infiltration, depleting neutrophil, and neutrophil adoptive transfer. Transcriptome sequencing and flow cytometry were performed to investigate the effects of HCC exosomes on the phenotype and function of neutrophils. The mobilization and apoptosis of neutrophils were evaluated by the Transwell experiment and Annexin V/7-AAD staining, respectively. Moreover, we detected the effects of HCC-exosome-reprogrammed neutrophils on T cells by flow cytometry. Next, we used the NF-κB pathway inhibitor JSH-23 and miR-362-5p inhibitor or mimic to determine the molecular mechanisms. Lastly, we constructed the miR-362-5p sponge to validate its targeted therapeutic potential.

**Results:** We found that HCC exosomes induced neutrophil infiltration and T-cell exhaustion in the livers of DEN/CCl_4_-induced HCC mice and promoted tumor progression. Blocking neutrophil infiltration and depleting neutrophils diminished these promotive effects of HCC exosomes. In addition, HCC exosome-reprogrammed neutrophils display proinflammatory and protumor phenotypes, and can directly induce T-cell exhaustion *in vitro*. The transfer of HCC exosome-reprogrammed neutrophils exacerbated tumor progression and induced T-cell exhaustion, as evidenced by the downregulation of IFN-γ and TNF-α, and the upregulation of PD-1 and Tim3 in T cells. Mechanistically, we found that HCC exosomes upregulate the expression of miR-362-5p in neutrophils and activate the NF-κB signaling pathway by targeting CYLD, promoting the survival and recruitment of neutrophils. In HCC mice, blocking miR-362-5p suppressed neutrophil infiltration, attenuated T-cell exhaustion, and suppressed HCC progression.

**Conclusions:** This study clarified the roles of HCC exosomes on neutrophil infiltration and reprogramming and identified a potential target miR-362-5p for HCC treatment.

## Introduction

Hepatocellular carcinoma (HCC) accounts for most liver cancer cases and related deaths worldwide. However, the first-line drugs for treating HCC approved by the FDA, sorafenib and lenvatinib, have only limited clinical efficacy 1. The accumulation of genetic mutations leads to heterogeneity in tumors, leading to significant obstacles for treatments targeting tumor cells 2. Therefore, targeting the relatively stable tumor microenvironment (TME) has become a promising therapeutic strategy. The TME is composed of tumor cells, stromal cells, immune cells, the extracellular matrix, cytokines, etc., and plays key roles in tumor occurrence, development, and prognosis. Notably, tumor-infiltrating immune cells, such as neutrophils, macrophages, and T cells, are reprogrammed to form an immunosuppressive microenvironment to promote tumor progression 3.

In the TME, neutrophils are one of the most abundant leukocytes, which are called tumor-associated neutrophils (TANs) and are associated with the progression and prognosis of various cancers, such as renal cell carcinoma 4, melanoma 5, and HCC 6. TANs exhibit high plasticity and heterogeneity 7, and the concepts of antitumor N1 and protumor N2 neutrophils were proposed to describe the different phenotypes of neutrophils 8. N1-type neutrophils kill tumor cells by releasing reactive oxygen species and reactive nitrogen species 9, 10 or activating the immune system 11, 12. In contrast, N2-type neutrophils promote tumor growth, metastasis, and angiogenesis and inhibit antitumor immunity.

Exosomes are a type of extracellular vesicle generated and secreted by cells through the endosomal pathway. Exosomes carry abundant parental cell components, such as nucleic acids, proteins, and lipids, which are important mediators of cell-to-cell communication 13. Increasing evidence has demonstrated that tumor-derived exosomes (TDEs) carry various information molecules and regulate TME features 14, 15. Importantly, TDEs play key roles in inducing TAN infiltration and immunosuppressive function. In colorectal cancer 16 and gastric cancer 17, TDEs promote the survival of TANs. Exosomes derived from lung cancer 18 and melanoma 19 mediate neutrophil infiltration into the premetastatic microenvironment to promote tumor metastasis. In addition, TDEs promote the formation of neutrophil extracellular traps, which participate in tumor metastasis, immune escape, and thrombosis 20, 21. However, the effects of HCC exosomes on neutrophils have not been elucidated.

In this study, we aimed to explore the effects of HCC exosomes on the infiltration and phenotype of neutrophils and to clarify the underlying molecular mechanisms involved in this process. Moreover, we elucidated the impact of HCC exosome-reprogrammed neutrophils on the antitumor immune response, especially the T-cell response. This study provides an experimental and theoretical basis for establishing a promising therapeutic strategy for HCC treatment based on the reprogramming of TANs.

## Material and Methods

### Cell lines

All cell lines Huh-7, Hepa 1-6, and HEK293T were purchased from the Cell Bank of Type Culture Collection of the Chinese Academy of Sciences (Shanghai, China). These cells were cultured in DMEM medium (Hyclone, Logan, Utah, USA) supplemented with 10% fetal bovine serum (FBS), 100 U/mL penicillin, and 100 mg/mL streptomycin and maintained in a 5% CO_2_ incubator at 37 °C.

### Animal experiments

C57BL/6J mice (6-8 weeks old) were purchased from Beijing HFK Bioscience Co., Ltd. (Beijing, China) and fed in specific pathogen-free conditions. All experiments were approved by the Institutional Animal Care and Use Committee of Shandong University and carried out following the Guide for the Care and Use of Laboratory Animals (Approval No.230105). To establish an induced HCC model, 2-week-old newborn C57BL/6J mice were intraperitoneally injected with diethylnitrosamine (DEN, 25 mg/kg, Sigma) weekly for a total of three times. Then carbon tetrachloride (CCl_4_, 0.5 μL/g, Fuyu chemical) was injected intraperitoneally weekly for a total of 16 times.

To explore the effects of HCC exosomes, Hepa 1-6 cell-exosomes (1 μg/g) were injected through the tail vein into the DEN/CCl_4_-induced HCC mice twice a week from 21-week-old to 24-week-old. To determine the role of neutrophils, anti-Ly6g blocking antibody (α-Ly6G, 200 μg per mice) or SB225002 (5 mg/kg, Selleck) was intraperitoneally injected into the DEN/CCl_4_-induced HCC mice twice a week from 21-week-old to 24-week-old, and mouse bone marrow-derived neutrophils (mBMDNs) (2×10^6^) treated with or without Hepa 1-6 cell-exosomes (40 μg/mL) were injected through the tail vein into the DEN/CCl_4_-induced HCC mice twice a week from 21-week-old to 24-week-old. To determine the role of miR-362-5p, miR-362-5p sponge (1 μg/g) constructed by General Biol Co., Ltd (Anhui, China) was targeted to the liver through hydrodynamic injection via tail vein in the DEN/CCl_4_-induced HCC mice once two weeks from 5-week-old to 24-week-old. At the time of euthanasia, we recorded the ratio of liver and body weight and the number of tumor nodules, and stained liver tissues with Hematoxylin-eosin to evaluate tumor progression. Mice were randomly divided into each group. Each group contains 6 mice for better statistical power.

### Exosome isolation

Ultracentrifugation was used to isolate exosomes as previously described 22. Briefly, supernatants from Huh-7 or Hepa 1-6 cells with over 95% viability were collected. To remove dead cells, cell debris, and larger vesicles, collected supernatants were sequentially centrifuged at 300 g for 10 min, 2000 g for 20 min, and 16500 g for 30 min at 4 °C. Then, the supernatant was filtrated through a 0.22 μm filter and ultracentrifuged at 120000 g for 1.5 h. The exosome pellets were accumulated at the bottom of the centrifuge tube. Then wash in a large volume of phosphate-buffered saline (1×PBS) and ultracentrifuge at 120000 g for 1.5 h at 4 °C. Lastly, resuspended exosomes in 1×PBS and stored at -80 °C for further use. Ultracentrifugation was carried out through L-100XP Ultracentrifuge (Beckman Coulter, USA). The protein content of the exosomes was measured by the BCA protein assay kit (P0010S, Beyotime).

### Western blotting analysis

Cells were lysed with RIPA lysis buffer (P0013B, Beyotime) for 10 min and centrifuged at 12000 g for 10 min at 4 °C. Supernatants were collected and added equal volume of 2 × loading buffer (AIWB-002, Affinibody). For the extraction of nuclear proteins, the Nuclear Protein Extraction Kit was used according to the manufacturer's instructions (R0050, Solarbio). The protein content was determined by the Enhanced BCA Protein Assay Kit (P0012S, Beyotime). Proteins were separated on sodium dodecyl sulfate-polyacrylamide gel electrophoresis (SDS-PAGE) and transferred onto polyvinylidene fluoride (PVDF) membranes. The membranes were blocked with 5% milk and incubated with specific antibodies listed in **Table S1**. Then, PVDF membranes were incubated with HRP-labelled goat anti-rabbit IgG (H+L) (A0208, Beyotime) or HRP-labelled goat anti-mouse IgG (H+L) (A0216; Beyotime) at room temperature. The signals were detected by NcmECL Ultra Enhanced Chemiluminescent (P10100, NCM Biotech).

### Neutrophil isolation and treatment

For isolation of human neutrophils, peripheral blood samples were collected from healthy donors after obtaining written informed consent. This study was approved by the Research Ethics Committee of Qilu Hospital of Shandong University (Approval No. KYLL-2021(KS)-1034). Layer anti-coagulant whole blood on an equal volume of Polymorphprep (AS1114683, Axis-Shield PoC AS, Norway) in a centrifuge tube. Centrifuge it at 550 g for 30 min at room temperature to form two leukocyte bands. The lower cell band was harvested and washed in 1×PBS, followed by red blood cell (RBC) lysis. The purity of neutrophils was over 95%.

For isolation of mBMDNs, the femur and tibia of C57BL/6J mice were collected and the bone marrow cells were flushed out with a syringe. Wash with 1×PBS and then resuspend bone marrow cells using 45% Percoll (GE Healthcare, Uppsala, Sweden). Carefully layer 81% Percoll, 62% Percoll, 55% Percoll, 50% Percoll, and 45% Percoll cell suspensions in sequence, and centrifuge at 1600 g for 30 min at room temperature to obtain granulocytes between 81% Percoll and 62% Percoll, and purified neutrophils can be obtained using the Anti-Ly-6G MicroBeads UltraPure (130-120-337, Miltenyi Biotec, Germany) with a purity of over 95%.

Neutrophils were cultured in RPMI 1640 (Hyclone, Logan, Utah, USA) supplemented with 10% FBS, 100 U/mL penicillin, and 100 mg/mL streptomycin at a density of 2×10^6^ per mL and treated with or without HCC exosomes. For the use of inhibitors, neutrophils were pretreated with CXCR2 inhibitor SB225005 (400 nM, Selleck), NF-κB inhibitor JSH-23 (400 nM, Selleck), or TNF-α inhibitor Benpyrine (2 μM, Selleck) for 2 h. For the miRNA interference, neutrophils were transfected with miRNA inhibitors, mimic, or corresponding negative control (NC) by Lipofectamine™ 2000 (11668019, Invitrogen) for 4 h. Then, these neutrophils were cultured with new medium and treated with HCC exosomes. The miR-362-5p inhibitor, mimic, and corresponding negative control were synthesized by Tsingke Biotech Co., Ltd. (Beijing, China).

### Isolation of leukocytes from tissues in mice

Liver leukocytes were isolated as previously described 23. Briefly, the liver was cut into pieces and passed through a 200-μm nylon cell strainer and then centrifuged at 100 g for 1 min to remove hepatocytes. The supernatant was centrifuged at 400 g for 5 min, and the deposited cells were resuspended by 5 mL 40% Percoll. The liver leukocytes were harvested after centrifugation at 800 g for 25 min, followed by RBC lysis. For splenic leukocytes, spleen tissues were cut into pieces and passed through a 200-μm nylon cell strainer. After centrifuging at 400 g for 5 min, single-cell suspensions were subjected to RBC lysis. The peripheral blood leukocytes were isolated after RBC lysis of peripheral blood directly.

### Flow cytometry

For the analysis of cell surface markers, single-cell suspensions were blocked with rat serum for 30 min and stained with fluorochrome-conjugated antibodies at 4 ˚C for 1 h. For the intracellular cytokines staining, cells were pre-treated with 1 μg/mL ionomycin (56092-81-0, MedChemExpree) and 50 ng/mL PMA (HY-18739, MedChemExpree) for 4 h in the presence of 1 μL/mL Brefeldin A (420601, BioLegend) for the last 3 h. After blocking with rat serum and staining with antibodies targeting membrane-expressing molecules, cells were fixed by 1% paraformaldehyde, permeabilized by the Permeabilization wash buffer (40403ES64, Yeasen), and stained with antibodies. Data were collected using a BD FACSymphony A3 or BD FACSCelesta system (BD Biosciences) and analyzed with FlowJo software (FlowJo, LLC, Ashland, OR, USA). The antibodies used are listed in **Table S2**.

### RNA sequencing

Human neutrophils from peripheral blood were treated with or without 40 μg/mL Huh-7 cell-exosomes for 12 h, and total mRNA was extracted using TRIzon reagent (CW0580, CWBIO). The mRNA-seq libraries were prepared following next-generation sequencing (NGS) protocols by Shanghai Personal Biotechnology Co., Ltd. (Shanghai, China) using the Illumina NovaSeq system. The datasets are available in the Bioproject database with the primary accession code PRJNA1133903.

### RNA extraction and quantitative real-time PCR (RT-qPCR)

Total RNA was extracted using TRIzon reagent (CWBIO, CW0580) and then used to synthesize cDNA using HiFiScript cDNA Synthesis Kit (CWBIO, CW2569) or the miRNA cDNA synthesis kit (CWBIO, CW2141). RT-qPCR was performed using the UltraSYBR one-step RT-qPCR kit (CWBIO, CW0659S) or miRNA qPCR assay kit (CWBIO, CW2142), respectively according to the manufacturer's instructions. The sequences of primers used listed in **Table S3** were synthesized by Tsingke Biotech Co., Ltd. (Beijing, China).

### Luciferase reporter gene assay

Firstly, we constructed a partial sequence in 3'-UTR of CYLD into the pmiRGLO vector (BR377, Fenghuishengwu) named wild-type CYLD plasmids. Then, the predicted miR-362-5p binding sites were mutated to obtain mutant CYLD plasmids. Primer sequences used for plasmid construction are listed in **Table S4**. Next, HEK293T cells were plated at a density of 8×10^3^ cells in a 96-well plate. After cell adherence, cells were co-transfected with NC or mimic and pmirGLO, wild-type CYLD plasmids, or mutant CYLD plasmids by Lipofectamine^TM^ 2000 (11668027, Invitrogen). After 48 h, firefly luciferase and renilla luciferase were detected using the Dual-Lumi™ luciferase assay kit (RG088S, Beyotime) according to the manufacturer's instructions using a microplate reader (Bio-Rad, Hercules, CA, USA).

### Statistical analysis

GraphPad Software Prism 9.0 (San Diego, CA, USA) was used for statistical analysis. The homogeneity of variance of data is detected by the Levene test. Two-tailed unpaired or paired t-tests or one-way ANOVA were performed to compare differences between different groups. Statistically significant differences were set at *p < 0.05, **p < 0.01, ***p < 0.001, ****p < 0.0001.

## Results

### HCC exosomes induce hepatic neutrophil accumulation and T-cell exhaustion and promote HCC progression

**Figure 1:**
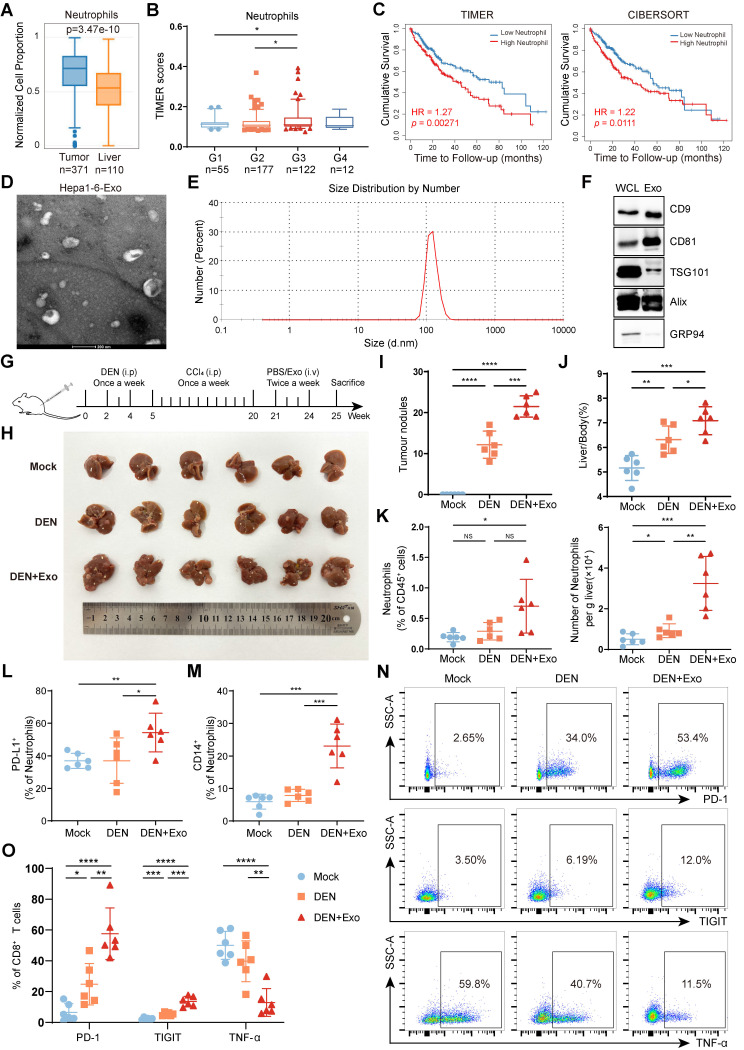
** HCC exosomes induce the hepatic accumulation of neutrophils, T-cell exhaustion, and promote HCC progression.** (**A**) Infiltration of neutrophils in liver cancer or normal liver was determined by using the GEPIA2021 database. (**B**) Infiltration of neutrophils in different stages of liver cancer was determined by using Assistant for Clinical Bioinformatics. (**C**) Patients with liver cancer were divided into 2 groups according to neutrophil infiltration scores calculated by TIMER or CIBERSORT, and the overall survival of these patients was determined by using TIMER2.0. (**D-F**) Hepa 1-6 cell-exosomes were isolated by ultracentrifugation and identified by transmission electron microscopy (**D**), particle size analyzer (**E**), and western blotting (**F**). (**G**) Schematic diagram of the treatment regimen with Hepa 1-6 cell-exosomes (1 μg/g). HCC was induced by DEN (25 mg/kg) and CCl_4_ (0.5 μL/g). (**H-K**) Representative liver images (**H**), the number of tumor nodules (**I**), and the liver/body weight (**J**) of mice (n = 6). The infiltration of neutrophils in the liver was determined by flow cytometry (**K**). (**L, M**) The proportion of PD-L1^+^ neutrophils (**L**) and CD14^+^ neutrophils (**M**) in the liver were analyzed by flow cytometry. (**N, O**) Flow cytometry was performed to analyze the expression of PD-1, TIGIT, and TNF-α on liver-infiltrating CD8^+^ T cells in mice. WCL, whole cell lysate; Exo, exosomes; DEN, DEN/CCl_4_-induced HCC mice; Mock, healthy mice. Data are presented as mean ± S.D. from at least three independent experiments. *p < 0.05, **p < 0.01, ***p < 0.001, and ****p < 0.0001.

To evaluate the correlation between TANs and HCC progression, we analyzed data from the TCGA database. We found that neutrophils extensively accumulated in liver cancer tissues (**Figure 1A**) and the number of TANs increased with the stage of tumor progression (**Figure 1B**). More importantly, high infiltration of neutrophils was associated with a poor prognosis in liver cancer patients (**Figure 1C**).

Owing to their high plasticity, neutrophils are usually reprogrammed by tumor-derived factors in the TME. TDEs carry rich parental cell components to mediate immune cell reprogramming. To clarify the role of HCC exosomes in neutrophil reprogramming and tumor progression, exosomes were extracted from murine Hepa 1-6 cells via ultracentrifugation and exhibited a typical tea tray-like morphology (**Figure 1D**) and 50-200 nm particle size (**Figure 1E**). Western blotting was performed to further confirm the successful isolation of HCC exosomes according to positive or negative markers of exosomes (**Figure 1F**). Subsequently, Hepa 1-6 cell-exosomes were injected through the tail vein into DEN/CCl_4_-induced HCC mice (**Figure 1G**). Compared to healthy mice (Mock), DEN/CCl_4_ treatment induced tumor nodule formation and increased the liver/body weight ratio, and HCC exosomes further augmented HCC progression (**Figure 1H‒J**). Notably, neutrophil infiltration was increased in the livers of DEN/CCl_4_-induced HCC mice, which was intensified by HCC exosome treatment (**Figure 1K, S1A**). Simultaneously, PD-L1^+^ neutrophils and CD14^+^ neutrophils that are associated with T-cell exhaustion 24 were enriched in liver tissues from HCC mice treated by HCC exosomes (**Figure 1L-M, S1B-C**).

Given the important role of TME remodeling, especially T-cell dysfunction in regulating tumor progression, we analyzed the effects of HCC exosome treatment on the liver-infiltrating T cells. Although there was no obvious influence on the number of liver-infiltrating T cells (**Figure S1D-F**), HCC exosomes significantly upregulated the expression of PD-1 and TIGIT and downregulated the expression of TNF-α on CD8^+^ T cells (**Figure 1N-O**) and CD4^+^ T cells (**Figure S1G-H**). These data indicate that HCC exosomes can promote the infiltration of neutrophils and T-cell exhaustion in the liver and exacerbate HCC progression.

### Depletion of neutrophils alleviates HCC exosome-induced T-cell exhaustion and tumor progression

**Figure 2:**
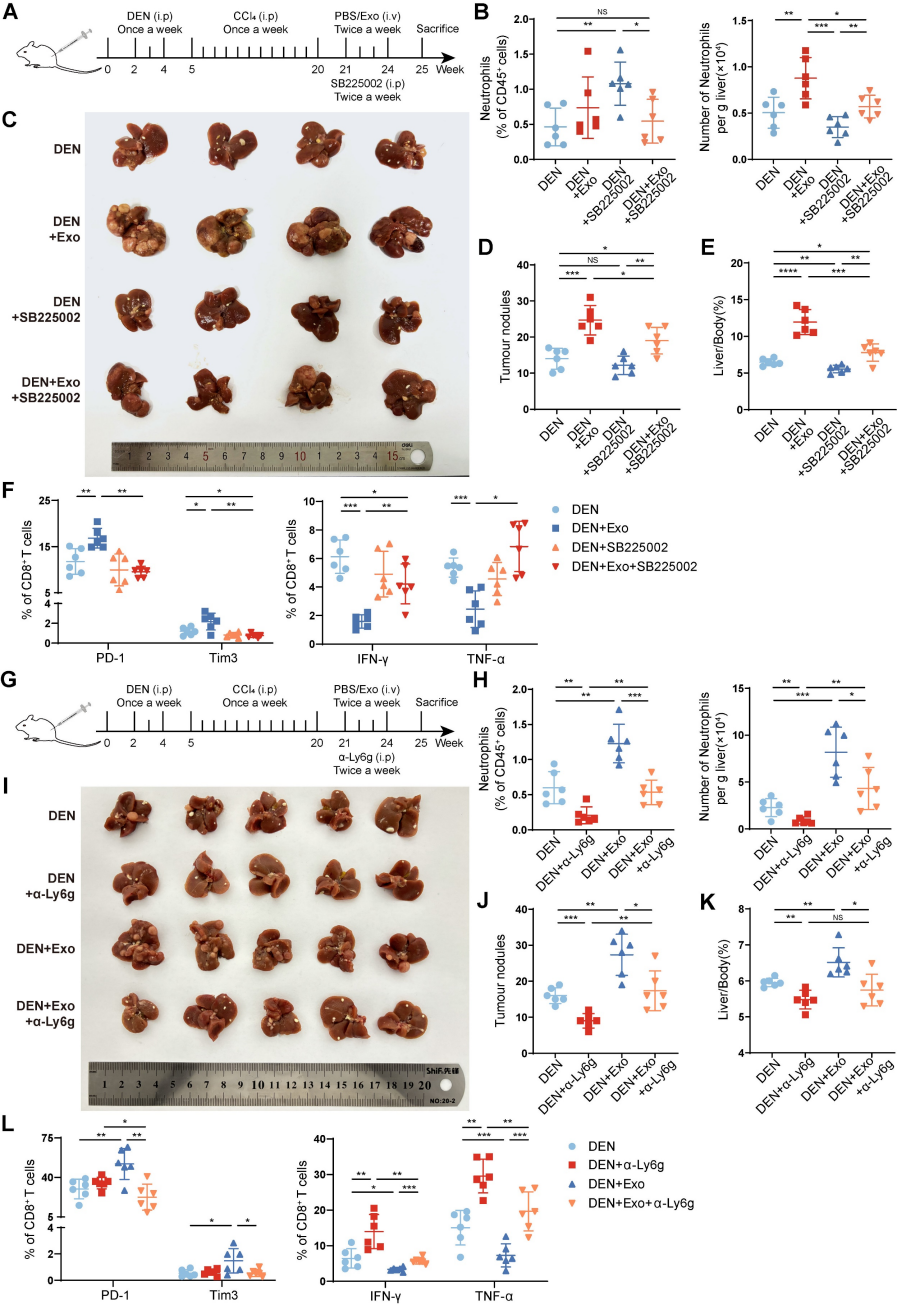
** Depletion of neutrophils alleviates HCC exosome-induced T-cell exhaustion and tumor progression.** (**A**) Schematic diagram of the treatment regimen with Hepa 1-6 cell-exosomes (1 μg/g), SB225002 (5 mg/kg), or the combination. (**B**) The infiltration of neutrophils was determined by flow cytometry (n = 6). (**C-E**) Representative liver images (**C**), the number of tumor nodules (**D**), and the liver/body weight (**E**) of mice (n = 6). (**F**) Flow cytometry was performed to analyze the expression of PD-1, Tim3, IFN-γ, and TNF-α on liver-infiltrating CD8^+^ T cells in mice. (**G**) Schematic diagram of the treatment regimen with Hepa 1-6 cell-exosomes (1 μg/g), α-Ly6g (200 μg), or the combination. (**H**) The infiltration of neutrophils was determined by flow cytometry (n = 6). (**I-K**) Representative liver images (**I**), the number of tumor nodules (**J**), and the liver/body weight (**K**) of mice (n = 6). (**L**) Flow cytometry was performed to analyze the expression of PD-1, Tim3, IFN-γ, and TNF-α on liver-infiltrating CD8^+^ T cells in mice. DEN, DEN/CCl_4_-induced HCC mice; Exo, exosomes; α-Ly6g, anti-Ly6g blocking antibody. Data are presented as mean ± S.D. from at least three independent experiments. *p < 0.05, **p < 0.01, ***p < 0.001, and ****p < 0.0001.

It has been verified that TANs can suppress T-cell function. To clarify the role of liver-infiltrating neutrophils in T-cell exhaustion and HCC progression induced by HCC exosomes, SB225002, the inhibitor of CXCR2 which is the major chemokine receptor of neutrophils, was used to block neutrophil infiltration (**Figure 2A**) 25, 26. The results showed that SB225002 treatment attenuated the promotive effect of HCC exosomes on the hepatic infiltration of neutrophils (**Figure 2B**). Notably, SB225002 inhibited HCC progression and reversed the protumor effect of HCC exosomes, decreasing the number of tumor nodules and the liver/body weight ratio (**Figure 2C-E**). Meanwhile, the effects of HCC exosomes on the induction of CD8^+^ T-cell (**Figure 2F, S2A**) and CD4^+^ T-cell (**Figure S2B-C**) exhaustion could be reversed by SB225002 treatment, as evidenced by the restoration of effector molecules IFN-γ and TNF-α, and exhaustion molecules PD-1 and Tim3.

To avoid the potential off-target effects of CXCR2 inhibitor SB225002, further, we used anti-Ly6g blocking antibody (α-Ly6g) to deplete neutrophils in DEN/CCl_4_-induced HCC mice (**Figure 2G-H**). Similar to SB225002 treatment, α-Ly6g treatment significantly impeded the protumor effect of HCC exosomes (**Figure 2I-K**), accompanied with the recovery of CD8^+^ T cells (**Figure 2L, S2D**) and CD4^+^ T cells (**Figure S2E-F**) from exhaustion induced by HCC exosomes. These results indicate that the liver accumulation of neutrophils contributes to HCC exosome-induced T-cell exhaustion and HCC progression.

### HCC exosomes induced migration, survival, and protumor polarization of neutrophils *in vitro*

**Figure 3:**
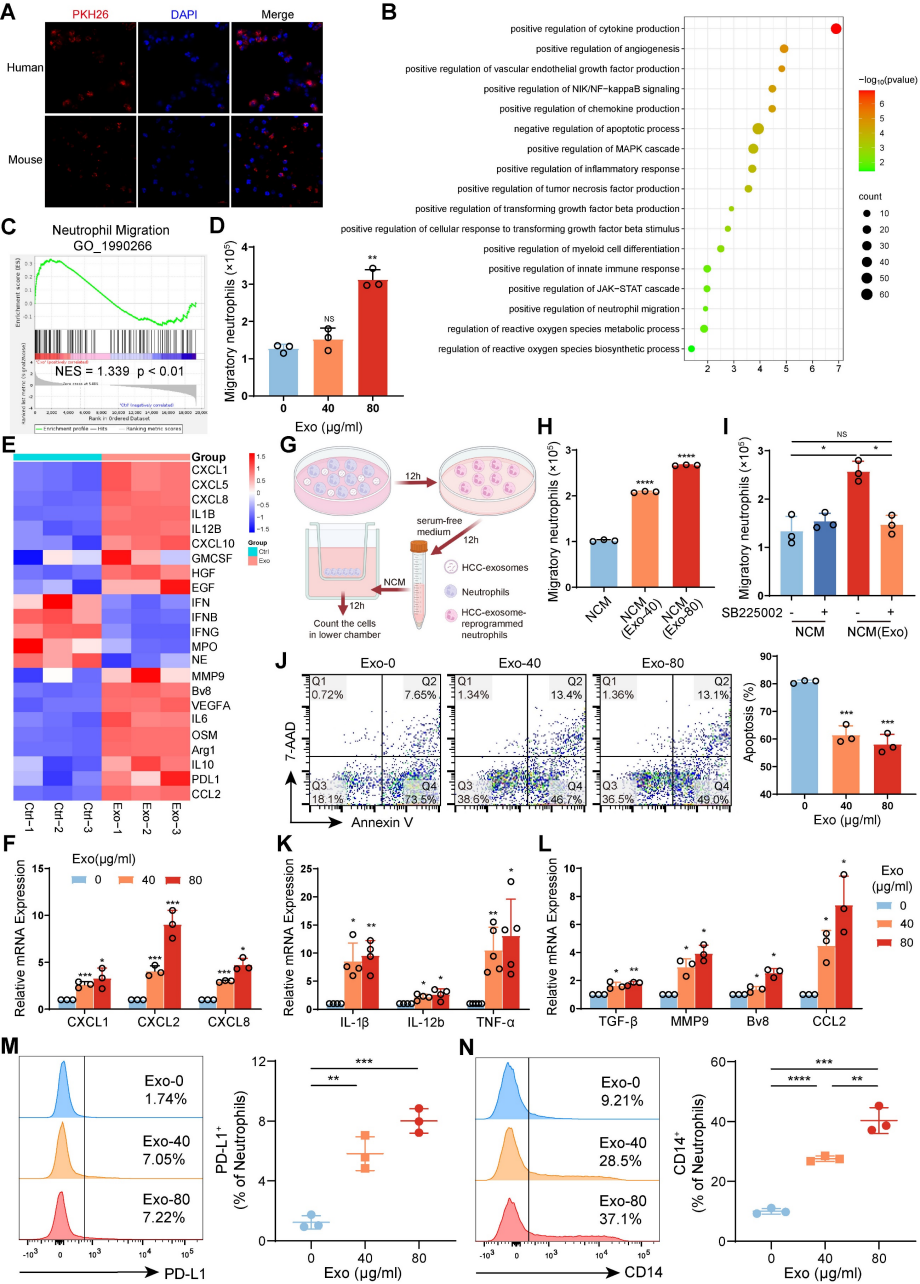
** HCC exosomes induced migration, survival, and protumor polarization of neutrophils *in vitro*.** (**A**) Neutrophils from human peripheral blood and mBMDNs were incubated with PKH26-labeled HCC exosomes from Huh7 and Hepa1-6 cells respectively for 4 h to analyze the uptake of HCC exosomes by neutrophils. (**B**) GO enrichment analysis of differentially expressed genes in human neutrophils treated with or without Huh-7 cell-exosomes (40 μg/mL) for 12 h (n = 3). (**C**) GSEA plot showing the enrichment scores for neutrophil migration. (**D**) Huh-7 cell-exosomes were added to recruit human neutrophils. After 12 h, the migration ability of neutrophils was determined by the number of neutrophils in the lower chamber. (**E**) Heatmap of chemotaxis and polarization-related genes in human neutrophils treated with or without Huh-7 cell-exosomes. (**F**) RT**-**qPCR assay was performed to detect the expression of CXCLs in human neutrophils treated with or without Huh-7 cell-exosomes. (**G**) Schematic diagram of the experiment to evaluate the influence of conditioned medium from neutrophils (NCM) or neutrophils treated with Huh-7 cell-exosomes (NCM(Exo)) on the naïve neutrophil migration. Created with BioRender.com. (**H, I**) NCM and NCM(Exo) were added to recruit neutrophils treated with or without SB225002 (400 nM). After 12 h, the migration ability of neutrophils was determined by the number of neutrophils in the lower chamber. (**J**) The apoptosis of human neutrophils treated with or without Huh-7 cell-exosomes for 12 h was detected by Annexin V/7AAD staining. (**K, L**) RT**-**qPCR was performed to detect the expression of inflammation (**K**) and polarization (**L**) related genes in human neutrophils treated with or without Huh-7 cell-exosomes. (**M, N**) Flow cytometry was performed to analyze the proportion of PD-L1^+^ mBMDNs and CD14^+^ mBMDNs treated with or without Hepa 1-6 cell-exosomes. Exo, exosomes. Data are presented as mean ± S.D. from at least three independent experiments. *p < 0.05, **p < 0.01, ***p < 0.001, and ****p < 0.0001.

To investigate the reprogramming effects of HCC exosomes on neutrophils, human neutrophils isolated from healthy donor peripheral blood or mouse bone marrow-derived neutrophils (mBMDNs) were incubated with human Huh-7 cell- or Hepa 1-6 cell-exosomes, respectively (**Figure S3A‒E**). By confocal microscopy, we observed that PKH26-labelled HCC exosomes could be taken up by human neutrophils and mBMDMs (**Figure 3A**). The transcriptional profiles of human neutrophils treated with or without HCC exosomes were subsequently analyzed via transcriptome sequencing (**Figure S4A**). Firstly, GO functional enrichment analysis and Gene set enrichment analysis (GSEA) revealed that HCC exosomes upregulated genes associated with the migration and survival of neutrophils (**Figure 3B-C**). The Transwell assay showed that the addition of HCC exosomes to the lower chamber promoted the migration of human neutrophils in the upper chamber (**Figure 3D**). RNA-seq and RT‒qPCR revealed that HCC exosomes upregulated the expression of CXCR2 ligands CXCL1, CXCL2, and CXCL8 in neutrophils (**Figure 3E-F**). Therefore, we analyzed the chemotactic effect of HCC exosome-treated neutrophils (**Figure 3G**). We found that the supernatant from HCC exosome-treated neutrophils had an enhanced chemotactic effect on naïve neutrophils (**Figure 3H**), which could be blocked by SB225002 (**Figure 3I**). Similarly, the migration and chemotaxis abilities of mBMDNs increased in a CXCL/CXCR2-dependent manner after Hepa 1-6 cell-exosomes treatment (**Figure S4B-E**). The physiological half-life of neutrophils is very short, but HCC exosomes inhibit neutrophil apoptosis (**Figure 3J, S4F**).

Secondly, GO functional enrichment analysis revealed that HCC exosomes increased the mRNA levels of inflammatory factors, chemokines, and angiogenic factors in neutrophils. The production and response of TNF in HCC exosome-treated neutrophils was enhanced, accompanied by the activation of the NF-κB and JAK-STAT signaling pathways, indicating a notable proinflammatory phenotype (**Figure 3B**).

Also, the transcriptome sequencing revealed that HCC exosome-reprogrammed neutrophils display N2 protumor phenotype with high expression of immunosuppressive genes, proangiogenic genes, and tumor metastasis-promoting genes (**Figure 3E**), and low expression of genes related to antitumor functions, such as cytotoxicity, respiratory burst, degranulation, or antigen presentation (**Figure S4G**). These results were partly confirmed by RT‒qPCR (**Figure 3K-L, S4H-I**). Similar to *in vivo* results, HCC exosomes could dose-dependently induce the expression of PD-L1 and CD14 on mBMDNs *in vitro* (**Figure 3M-N**). All data above indicate that HCC exosomes enhance the migration and survival of neutrophils in the TME and that HCC exosome-reprogrammed neutrophils present a proinflammatory and protumor N2 phenotype.

### HCC exosome-reprogrammed neutrophils induce T-cell exhaustion and promote tumor progression

**Figure 4:**
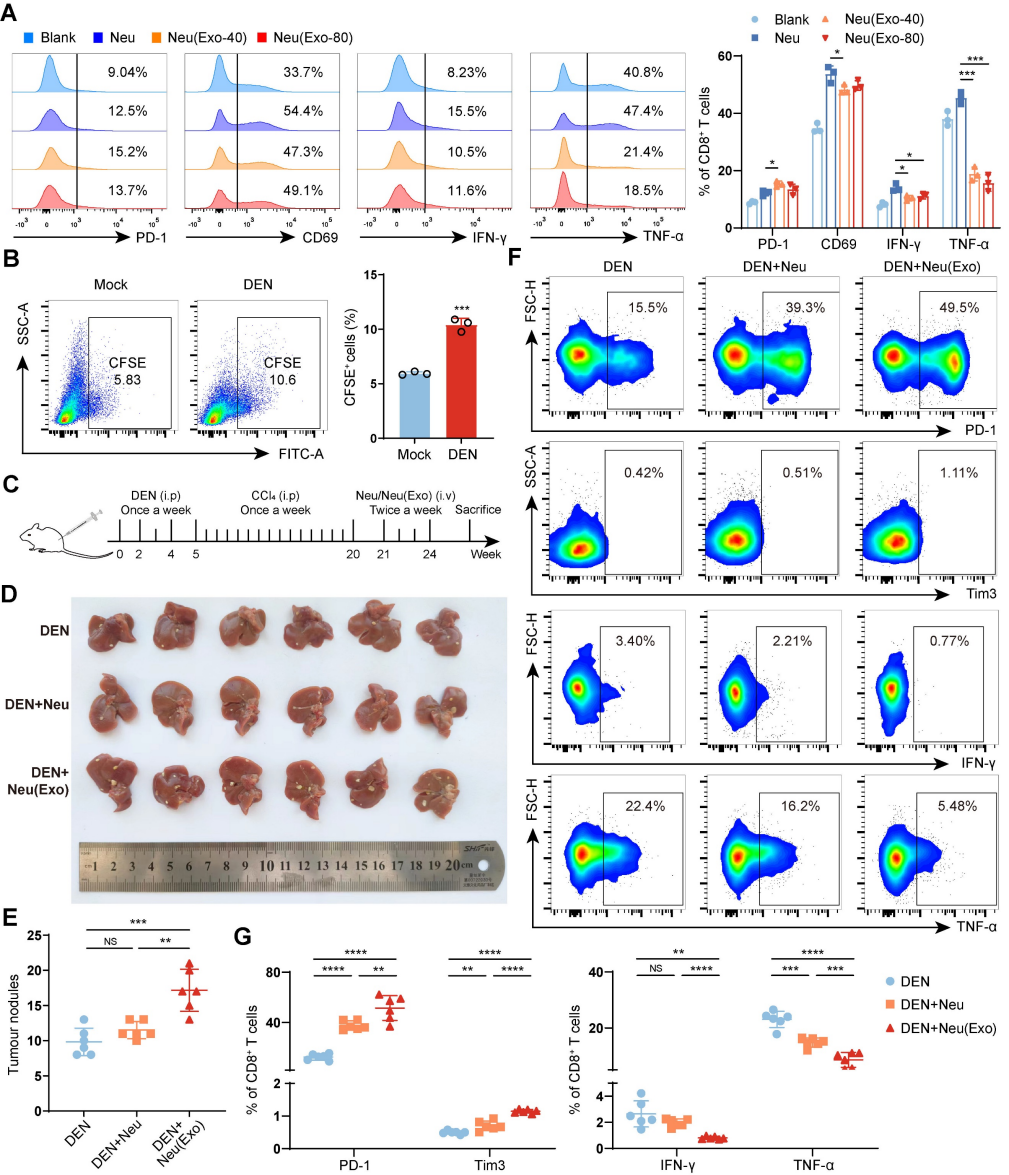
** HCC exosome-reprogrammed neutrophils induce CD8^+^ T cell exhaustion and promote tumor progression.** (**A**) T cells isolated from the spleen of healthy mice were co-cultured with mBMDNs treated with or without Hepa 1-6 cell-exosomes for 48 h. Flow cytometry was performed to analyze the expression of PD-1, CD69, IFN-γ, and TNF-α on CD8^+^ T cells. (**B**) CFSE-labelled mBMDNs (2×10^6^) were transferred into healthy mice (Mock) or DEN/CCL_4_-induced HCC mice via the tail vein. After 24 h, leukocytes were harvested from the liver to analyze the proportion of CFSE^+^ cells using flow cytometry. (**C**) Schematic diagram of adoptive transferring mBMDNs (2×10^6^) treated with or without Hepa 1-6 cell-exosomes (40 μg/mL). (**D, E**) Representative liver images (**D**) and the number of tumor nodules (**E**) of mice (n = 6). (**F, G**) Flow cytometry was performed to analyze the expression of PD-1, Tim3, IFN-γ, and TNF-α on liver-infiltrating CD8^+^ T cells. Mock, healthy mice; DEN, DEN/CCl_4_-induced HCC mice; Neu, mBMDNs; Neu(Exo), mBMDNs treated with Hepa 1-6 cell-exosomes; Exo, exosomes. Data are presented as mean ± S.D. from at least three independent experiments. *p < 0.05, **p < 0.01, ***p < 0.001, and ****p < 0.0001.

To confirm whether HCC exosome-reprogrammed neutrophils directly induce T-cell exhaustion and tumor progression, splenic T cells isolated from healthy mice (**Figure S5A**) were cocultured with mBMDNs reprogrammed with or without HCC exosomes *in vitro*. The results showed that HCC exosome-reprogrammed mBMDNs could upregulate the expression of PD-1, but suppress the expression of CD69, and the production of IFN-γ and TNF-α in CD8^+^ T cells (**Figure 4A**) and CD4^+^ T cells (**Figure S5B**). Next, the protumor function of HCC exosome-reprogrammed neutrophils *in vivo* was determined via mBMDN transfer experiments. First, CFSE-labelled mBMDNs were transferred via the tail veins of the mice, and the results verified that the transferred mBMDNs could migrate to the livers of the mice, especially to the livers of the DEN/CCl_4_-induced HCC mice (**Figure 4B**). Then, mBMDNs treated with or without Hepa1-6 cell exosomes were injected through the tail vein into DEN/CCl_4_-induced HCC mice (**Figure 4C**). Compared to naïve mBMDNs, HCC exosome-reprogrammed mBMDNs significantly promoted the formation of tumor nodules (**Figure 4D-E**), increased the expression of PD-1 and Tim3, and reduced the expression of IFN-γ and TNF-α in liver-infiltrating CD8^+^ T cells (**Figure 4F-G**) and CD4^+^ T cells (**Figure S5C-D**). These results demonstrate that HCC exosome-reprogrammed neutrophils can induce T-cell exhaustion and promote tumor progression *in vivo*.

### HCC exosomes reprogram neutrophils by activating the NF-κB signaling pathway

**Figure 5:**
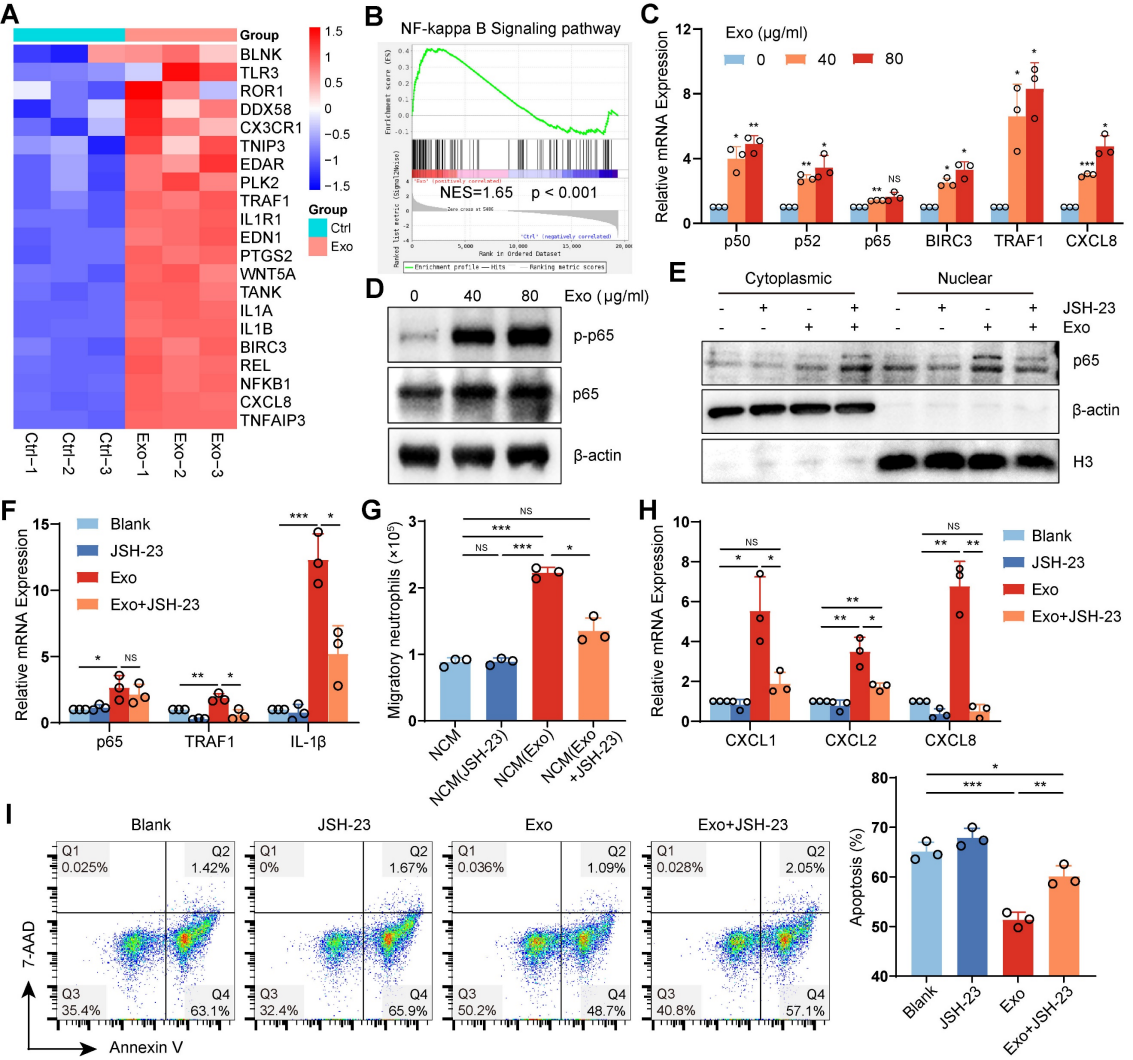
** HCC exosomes reprogram human neutrophils by activating the NF-κB signaling pathway.** (**A**) Heatmap of NF-κB signaling-related genes differentially expressed in human neutrophils treated with or without Huh-7 cell-exosomes. (**B**) GSEA plot showing the enrichment scores for NF-κB pathway in human neutrophils treated with Huh-7 cell-exosomes. (**C, D**) RT-qPCR assay and western blotting assay were performed to detect the activation of the NF-κB signaling pathway in human neutrophils treated with or without Huh-7 cell-exosomes. (**E-I**) Human neutrophils were treated with JSH-23 (400 nM), Huh-7 cell-exosomes (40 μg/mL), or the combination. Exosomes were added 2 h post JSH-23 treatment. Western blotting assay was performed to detect the levels of p65 in the cytoplasm and nucleus of neutrophils (**E**). RT-qPCR assay was performed to detect the mRNA levels of NF-κB signaling pathway-related genes (**F**). Conditioned mediums from these neutrophils were added to recruit naive neutrophils (**G**). RT-qPCR assay was performed to detect the expression of chemokines CXCLs in neutrophils (**H**). The apoptosis of neutrophils was detected by Annexin V/7-AAD staining (**I**). Exo, exosomes; NCM, conditioned medium from neutrophils. Data are presented as mean ± S.D. from at least three independent experiments. *p < 0.05, **p < 0.01, ***p < 0.001, and ****p < 0.0001.

NF-κB is a key transcription factor involved in innate immunity and inflammation. RNA sequencing and RT‒qPCR revealed that the NF-κB pathway was significantly activated in HCC exosome-reprogrammed neutrophils (**Figure 5A‒C**). Western blotting revealed that HCC exosomes promoted the phosphorylation (**Figure 5D**) and nuclear translocation of p65 in neutrophils (**Figure 5E**), which could be suppressed by the NF-κB pathway inhibitor JSH-23 (**Figure 5E-F**). Notably, JSH-23 treatment attenuated the ability of HCC exosomes to promote neutrophil chemotaxis (**Figure 5G, S6A**), accompanied by the downregulation of the chemokine CXCLs in neutrophils (**Figure 5H**), and prevented the inhibitory effect of HCC exosomes on neutrophil apoptosis (**Figure 5I, S6B**).

TNF-α is a canonical upstream signal molecule that activates the NF-κB pathway. The GSEA result indicated that HCC exosomes significantly activated the TNF pathway in neutrophils (**Figure S6C**). However, TNF-α inhibitor benpyrine did not influence the effects of HCC exosomes on the neutrophil chemotaxis (**Figure S6D**) or apoptosis (**Figure S6E**). These data suggest that HCC exosomes reprogram neutrophils by activating the NF-κB signaling pathway, which is independent of TNF-α/TNFR signal transduction.

### HCC exosome-induced miR-362-5p enrichment activates the NF-κB signaling pathway in neutrophils

**Figure 6:**
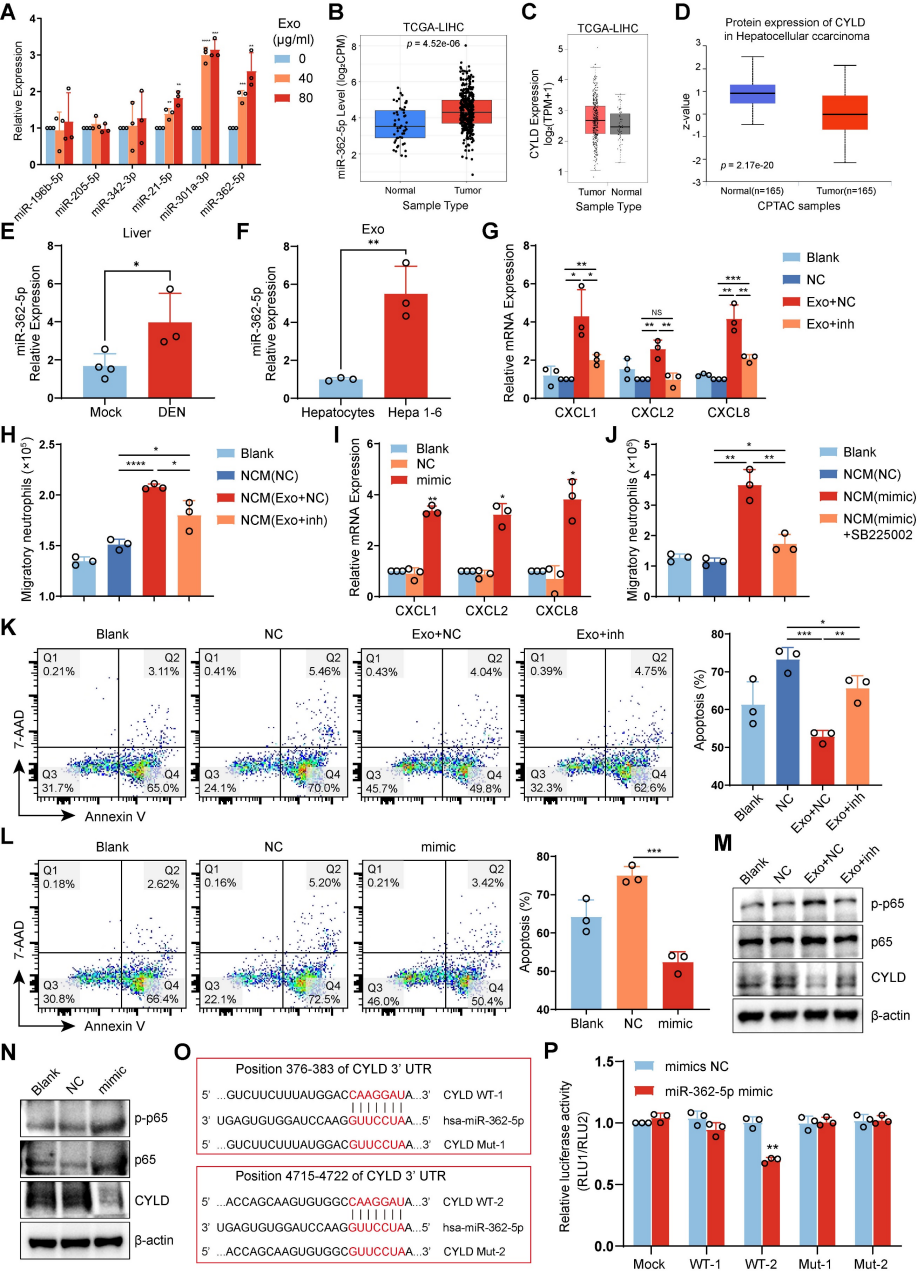
** HCC exosome-induced miR-362-5p enrichment activates the NF-κB signaling pathway in neutrophils.** (**A**) RT**-**qPCR assay was performed to detect the expression of miRNAs in human neutrophils treated with or without Huh-7 cell-exosomes. (**B**) The levels of has-miR-362-5p in liver cancer or normal liver were evaluated by using the CancerMIRNome database. (**C**) The mRNA levels of CYLD in liver cancer or normal liver were evaluated by using the GEPIA2 database. (**D**) The protein levels of CYLD in liver cancer or normal liver were evaluated by using the UALCAN database. (**E**) RT-qPCR was performed to detect the level of miR-362-5p in the liver of healthy mice and DEN/CCl4-induced HCC mice. (**F**) RT-qPCR was performed to detect the level of miR-362-5p in hepatocyte-exosomes from healthy mice and Hepa 1-6 cell-exosomes. (**G, H, K, M**) Human neutrophils were transfected with NC or miR-362-5p inhibitor for 4 h and then treated with Huh-7 cell-exosomes (40 μg/mL) for another 12 h. RT-qPCR assay was performed to detect the expression of chemokines CXCLs in neutrophils (**G**). The conditioned mediums as indicated were collected to recruit naïve neutrophils (**H**). The apoptosis of neutrophils was detected by Annexin V/7-AAD staining (**K**). Western Blotting was performed to detect the expression of CYLD and the activation of the NF-κB signaling pathway in neutrophils (**M**). (**I, J, L, N**) Human neutrophils were transfected with NC or miR-362-5p mimic for 12 h. RT**-**qPCR assay was performed to detect the expression of chemokines CXCLs in neutrophils (**I**). The conditioned mediums as indicated were collected to recruit neutrophils pretreated with or without SB225002 (400 nM) (**J**). The apoptosis of neutrophils was detected by Annexin V-7/AAD staining (**L**). Western Blotting was performed to detect the expression of CYLD and the activation of the NF-κB signaling pathway in neutrophils (**N**). (**O**) The binding sites of has-miR-362-5p on CYLD mRNA were predicted by TargetScan (www.targetscan.org), and the corresponding vector with wild-type (WT) CYLD or CYLD with mutant binding site (Mut) in pcDNA3.1 vector were constructed. (**P**) HEK293T cells were co-transfected with NC or mimic and pcDNA3.1, wild-type CYLD plasmids, or mutant CYLD plasmids, respectively. After 48 h, relative luciferase activity was detected using a microplate reader. DEN, DEN/CCl_4_ induced HCC mice; NC, negative control; inh, miR-362-5p inhibitor; Exo, exosomes; NCM, conditioned medium from neutrophils. Data are presented as mean ± S.D. from at least three independent experiments. *p < 0.05, **p < 0.01, ***p < 0.001, and ****p < 0.0001.

Various miRNAs, including miR-196b-5p, miR-205-5p, miR-342-3p, miR-21-5p, miR-301a-3p, and miR-362-5p, have been reported to activate the NF-κB pathway 27-32. To determine whether HCC exosomes activate the NF-κB signaling pathway in neutrophils through miRNA, we detected the levels of these miRNAs. We found that HCC exosomes upregulated miR-301a-3p and miR-362-5p levels in both human neutrophils and mBMDNs (**Figure 6A, S7A**). TCGA database analysis also revealed that miR-362-5p and miR-301a-3p were upregulated in liver cancer tissues compared with normal liver tissues (**Figure 6B, S7B**).

MiRNAs can inhibit the translation of target mRNAs. However, the expression of NF-κB repressing factor (NKRF), the reported target gene of miR-301a-3p, was upregulated at both the mRNA and protein levels in liver cancer tissues (**Figure S7C, D**). In comparison, CYLD lysine 63 deubiquitinase (CYLD), the reported target gene of miR-362-5p, showed no significant changes at the mRNA level but decreased at the protein level in liver cancer tissues (**Figure 6C, D**).

In addition, the levels of miR-362-5p in the livers of HCC mice were higher than in normal livers (**Figure 6E**). Importantly, compared with hepatocyte-exosomes from healthy mice, miR-362-5p levels significantly increased in Hepa 1-6 cell-exosomes (**Figure 6F**). CYLD is an upstream suppressor of NF-κB, so we speculated that miR-362-5p but not miR-301a-3p might be involved in regulating the NF-κB pathway in HCC exosome-reprogrammed neutrophils.

To confirm this speculation, we used miR-362-5p inhibitor or mimic to intervene in the effects of miR-362-5p on neutrophils. We found that the miR-362-5p inhibitor weakened HCC exosome-induced CXCL expression and the chemotactic effect on neutrophils (**Figure 6G-H, S7E**). Conversely, the effects of miR-362-5p mimic were similar to those of the HCC exosomes, increasing CXCL expression and the CXCL/CXCR2-dependent chemotactic effect on neutrophils (**Figure 6I-J, S7F**). Additionally, the miR-362-5p inhibitor attenuated the inhibitory effect of HCC exosomes on neutrophil apoptosis (**Figure 6K, S7G**), whereas the miR-362-5p mimic suppressed neutrophil apoptosis (**Figure 6L, S7H**). Therefore, miR-362-5p regulates the NF-κB pathway in HCC exosome-reprogrammed neutrophils.

Next, we assessed whether miR-362-5p regulates the activation of the NF-κB pathway in neutrophils by targeting CYLD. Western blotting revealed that HCC exosomes inhibited the expression of CYLD and activated the NF-κB pathway in neutrophils, and these effects were reversed by treatment with the miR-362-5p inhibitor (**Figure 6M**). Similar to HCC exosomes, miR-362-5p mimic suppressed the expression of CYLD and activated the NF-κB pathway in neutrophils (**Figure 6N**). The TargetScan database predicted that miR-362-5p might target two sites in the 3'-UTR of CYLD (**Figure 6O**). We found that miR-362-5p mimic could reduce the luciferase activity of only the WT2 vector, and this effect was attenuated by the mutation (Mut2) (**Figure 6P**), indicating that miR-362-5p mainly regulates the expression of CYLD by targeting the 4715‒4722 sequence of CYLD mRNA. These data suggest that HCC exosome-induced miR-362-5p activates the NF-κB pathway by blocking CYLD translation.

### Blocking miR-362-5p can reverse the protumor effect of neutrophils

**Figure 7:**
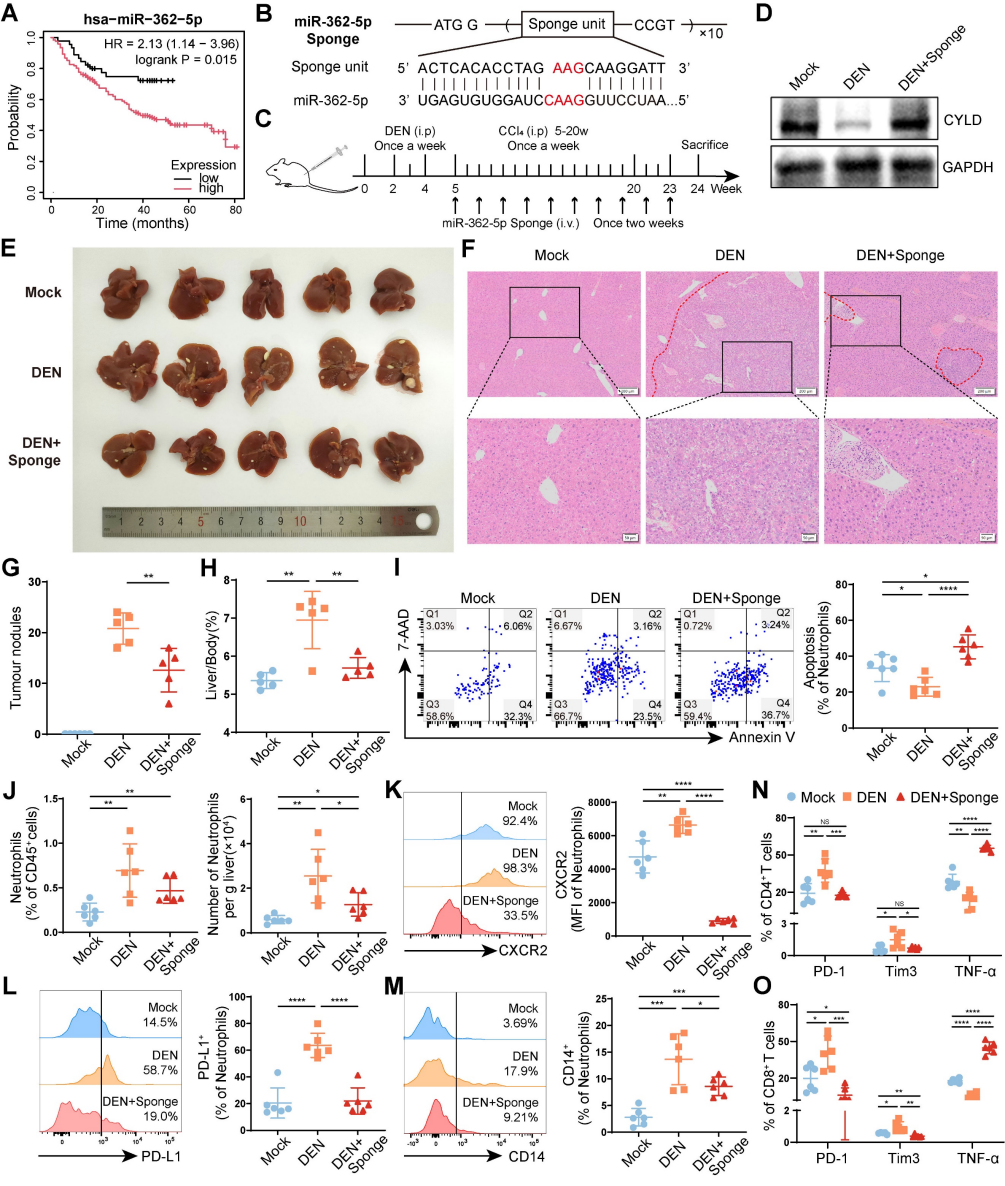
** Blocking miR-362-5p can reverse the protumor effects of neutrophils. (A**) The correlation of miR-362-5p expression and prognosis of patients with liver cancer. (**B**) Schematic diagram of the structure of miR-362-5p sponge. (**C**) Schematic diagram of the treatment regimen with miR-362-5p sponge (1 μg/g). (**D**) Western blotting was performed to detect CYLD expression in the liver tissues. (**E-H**) Representative liver images (**E**), hematoxylin and eosin staining liver images (**F**), the number of tumor nodules (**G**), and the liver/body weight (**H**) of mice (n = 6). (**I-O**) Leukocytes were harvested from the livers of mice. The apoptosis of neutrophils was detected by Annexin V/7-AAD staining (**I**), and the abundance of neutrophils (**J**) and the expression of CXCR2 (**K**), PD-L1 (**L**), and CD14 (**M**) on neutrophils were determined by flow cytometry. The expressions of PD-1, Tim3, and TNF-α on CD4^+^ T cells (**N**) and CD8^+^ T cells (**O**) of mice were determined by flow cytometry. Mock, healthy mice, DEN, DEN/CCl_4_-induced HCC mice. Data are presented as mean ± S.D. from at least three independent experiments. *p < 0.05, **p < 0.01, ***p < 0.001, and ****p < 0.0001.

The survival analysis revealed that higher miR-362-5p expression in tumors was correlated with poor prognosis of patients with liver cancer (**Figure 7A**). To explore whether blocking miR-362-5p can suppress HCC progression, we designed a tandem repeat sequence containing a miR-362-5p antisense fragment and incorporated it into a pcDNA3.1(-) myc-His-B vector; we named the resulting vector the miR-362-5p sponge (**Figure 7B**). Then, the miR-362-5p sponge was hydrodynamically injected into DEN/CCl_4_-induced HCC mice through the tail vein (**Figure 7C**). The level of CYLD was lower in the livers of HCC mice than in those of healthy mice (Mock), which was restored by the miR-362-5p sponge (**Figure 7D**). And we found that the miR-362-5p sponge inhibited HCC progression, reducing the number of tumor nodules (**Figure 7E-G**) and the liver/body weight ratio (**Figure 7H**). Meanwhile, compared with those in healthy mice (Mock), the proportion of apoptotic liver-infiltrating neutrophils was lower, and the overall number of liver-infiltrating neutrophils was higher in DEN/CCl_4_-induced HCC mice, which could be reversed by the treatment of the miR-362-5p sponge (**Figure 7I-J**). Moreover, the miR-362-5p sponge decreased neutrophil infiltration into the spleens (**Figure S8A**), but increased neutrophil infiltration into the peripheral blood (**Figure S8B**). Notably, the miR-362-5p sponge reduced the proportion of CXCR2^+^ neutrophils in the liver (**Figure 7K**), whereas enhanced in the peripheral blood (**Figure S8C**). These data indicate that the miR-362-5p sponge can promote neutrophil apoptosis and block the infiltration of circulating CXCR2^+^ neutrophils into the liver.

Finally, we evaluated the effects of the miR-362-5p sponge on the phenotypes of TANs and T cells. The miR-362-5p sponge inhibited the expression of PD-L1 and CD14 on neutrophils in the livers of DEN/CCl_4_-induced HCC mice (**Figure 7L-M**) and restored the function of hepatic CD4^+^ T cells (**Figure 7N, S8D**) and CD8^+^ T cells (**Figure 7O, S8E**), as indicated by downregulated PD-1 and Tim3 expression and upregulated TNF-α production. Similar changes were observed in splenic CD4^+^ T cells (**Figure S8F**) and CD8^+^ T cells (**Figure S8G**). These data suggest that the miR-362-5p sponge can suppress neutrophil infiltration and restore T-cell functions in the liver, preventing HCC progress.

## Discussion

Given that HCC exosomes may be important mediators in regulating TANs, we explored the roles and potential mechanisms of HCC exosomes in regulating TANs. In many kinds of tumors, TDEs directly or indirectly promote the infiltration of TANs. For example, Rab27a and Rab27b regulate the exocytosis of multivesicular bodies, leading to exosome secretion. Blockade of Rab27a or Rab27b reduces the infiltration of TANs in a mouse model of breast cancer or colorectal cancer, inhibiting tumor progression 33, 34. In this study, we analyzed the TCGA database and found that increased infiltration of neutrophils in liver cancer was positively correlated with poor prognosis. In addition, we found that HCC exosomes promoted the hepatic accumulation of neutrophils in DEN/CCl_4_-induced HCC mice, which was associated with T-cell exhaustion and tumor progression. These findings indicate that HCC exosomes can augment neutrophil infiltration in the liver and reprogram them to promote tumor progression.

The CXCL-CXCR1/2 axis is important for TAN infiltration 7. We demonstrated that HCC exosomes not only directly recruited neutrophils but also upregulated the expression of CXCL chemokines in neutrophils, which further recruited more neutrophils via the CXCL-CXCR1/2 axis. In addition, HCC cells may secrete chemokines to participate in neutrophil infiltration. We found that HCC exosomes did not significantly influence the expression of CXCL chemokines (**Figure S9A**), but HCC exosome-reprogrammed neutrophils upregulated the expression of CXCL1, CXCL2, and CXCL8 in HCC cells, forming a positive feedback loop to recruit more neutrophils (**Figure S9B**). Recent studies have shown that TDEs derived from colorectal cancer and gastric cancer cells transfer RNAs or the HMGB1 protein to maintain the survival of TANs 16, 17. We found that HCC exosomes also inhibited neutrophil apoptosis, which is more conducive to neutrophil infiltration and aggravates tumor progression.

In many kinds of tumors, TDEs can directly inhibit T-cell function 35-37. However, our previous study and several other studies have shown that HCC exosomes indirectly disturb T-cell function via the expression of PD-L1 on macrophages 38-40. TANs disturb T-cell functions through a variety of mechanisms, including PD-1/PD-L1 interaction 24, and the CD14^+^ neutrophil was recently shown to be associated with immunosuppressive function 41. In our study, we found that HCC exosomes promoted N2 polarization of neutrophils and induced the enrichment of PD-L1^+^ neutrophils and CD14^+^ neutrophils. Meanwhile, HCC exosome reprogrammed mBMDNs promoted T-cell exhaustion, as evidenced by high expression of PD-1 and Tim-3, and low production of IFN-γ and TNF-α. Therefore, the immunosuppressive function of neutrophils might be critical for inducing T-cell exhaustion and tumor progression. Many factors or pathways are associated with the immunosuppressive function of neutrophils, and the NF-κB and JAK/STAT3 signaling pathways have been reported to regulate PD-L1 expression 42, 43. However, neither NF-κB inhibitor JSH-23 nor STAT3 inhibitor BBI608 could reverse HCC exosome-induced upregulation of PD-L1 and CD14 on neutrophils (**Figure S10A-B**). In addition, neutrophil extracellular traps (NETs) can induce T-cell suppression and promote tumor progression 44, 45, but HCC exosomes did not influence the formation of NETs (**Figure S10C**). Overall, the molecular mechanisms related to N2 polarization and immunosuppression of neutrophils deserve to be studied in depth.

To clarify the mechanism by which HCC exosomes regulate the migration, chemotaxis, and apoptosis of neutrophils, we performed transcriptome sequencing analysis and focused on the activating NF-κB pathway. NF-κB is a key transcription factor involved in innate immunity and inflammation, as well as the occurrence and development of tumors 46. Studies have shown that the activation of the NF-κB pathway is important for TDE-mediated regulation of TANs. TDEs activate the NF-κB pathway to maintain the survival of TANs in colorectal and gastric cancer and recruit neutrophils via promoting the secretion of IL-8 16, 17, a canonical downstream chemokine of the NF-κB pathway 47. Liwen Wang et al. identified protumor subsets TAN-1 and TAN-2 with the activation state of the NF-κB pathway in pancreatic cancer 48. Here, we confirmed that the inhibition of the NF-κB pathway with JSH-23 could reverse the effects of HCC exosomes on neutrophils, including effects on neutrophil apoptosis, migration, and chemotaxis. Therefore, HCC exosomes reprogram neutrophils partly by activating the NF-κB signaling pathway.

NF-κB signaling pathway can be regulated by various signals, like TNF/TNFR and miRNAs. Transcriptome sequencing analysis showed that HCC exosomes significantly activated the TNF signaling pathway in neutrophils. However, the TNF-α inhibitor Benpyrine did not influence the effects of HCC exosomes on the chemotaxis or apoptosis of neutrophils. Whereas, by targeting CYLD, miR-362-5p could activate the NF-κB signaling pathway and influence the chemotaxis and apoptosis of neutrophils. The activation of the NF-κB pathway involves the attachment of K63-linked ubiquitin chains to its upstream factors, which facilitates protein-protein interactions. In contrast, CYLD can deubiquitinate the upstream regulatory factors of NF-κB, such as tumor necrosis factor receptor-associated factor (TRAF) 2, TRAF6, and NEMO 49-51, preventing excessive activation of NF-κB through negative feedback 52. Compared to healthy donors, miR-362-5p was highly expressed in the circulation of patients with various cancers (**Figure S11**), and higher miR-362-5p in tumors was associated with poor prognosis for patients with liver cancer. Previous studies have shown that miR-362-5p can directly regulate tumor cells, promoting the proliferation, migration, and invasion of liver cancer cells 32. Our findings complement the fact that in HCC, miR-362-5p regulates neutrophil function and promotes tumor progression by targeting CYLD. Exosomes are important delivery carriers of miRNAs, and we confirmed that miR-362-5p was enriched in HCC exosomes, thus, we speculated that miR-362-5p enrichment in neutrophils may be through the delivery of TDEs. However, we have not excluded that HCC exosomes activate relevant signals within neutrophils to make them express higher levels of miR-362-5p, which is worthy of further exploration.

NK cells are another tumor-killing immune cells and have been reported to be regulated by TANs 53, 54. In DEN/CCl_4_-induced HCC mice, we observed HCC exosome treatment did not affect the number of liver-infiltrating NK cells (**Figure S12A**), but induced NK cell functional exhaustion, as evidenced by the downregulation of TNF-α and the upregulation of PD-1 and TIGIT (**Figure S12B-C**). However, the depletion of neutrophils by α-Ly6g treatment could not reverse the functional exhaustion of NK cells (**Figure S12D-E**), indicating HCC exosomes disturb NK cells through pathways other than neutrophils, and the related mechanisms need to be investigated in the future.

Based on the above, we verified the role of miR-362-5p in DEN/CCl_4_-induced HCC mice. The results showed that the miR-362-5p sponge blocked neutrophil infiltration and promoted its apoptosis, and reduced PD-L1^+^ neutrophils and CD14^+^ neutrophils, alleviating T-cell exhaustion. Given that miR-362-5p is also able to directly promote the proliferation and migration of HCC cells, we think targeting miR-362-5p can inhibit the malignant potential of tumor cells, and simultaneously suppress TAN infiltration and reprogramming. Furthermore, TANs mediate chemotherapy and immunotherapy resistance 6, 55, 56. Therefore, we speculated that targeting miR-362-5p combined with sorafenib or immune checkpoint blockade therapy might exhibit a synergistic effect and is expected to reverse TAN infiltration-mediated resistance to chemotherapy and immunotherapy.

In conclusion, HCC exosome-induced miR-362-5p enrichment in neutrophils activates the NF-κB pathway by targeting the 3'-UTR of CYLD, which promotes the survival and chemotaxis of neutrophils. In addition, HCC exosomes induced a protumor phenotype of neutrophils, resulting in T-cell exhaustion and tumor progression. Blocking miR-362-5p showed strong anti-tumor effects with improved tumor microenvironment, which might be a potential target for HCC treatment based on neutrophil reprogramming.

## Supplementary Material

Supplementary materials and methods, figures.

## Abbreviations

3'-UTR: 3'-untranslated region
CCl_4_: carbon tetrachloride
CXCLs: C-X-C motif chemokine ligands
CXCR2: C-X-C motif chemokine receptor 2
CYLD: CYLD lysine 63 deubiquitinase
DEN: N-Nitrosodiethylamine
GO: gene ontology
GSEA: gene set enrichment analysis
IFN-γ: interferon gamma
JAK-STAT: janus kinase-signal transduction and transcription activation
mBMDNs: mouse bone marrow-derived neutrophils
Mut: mutant
NETs: neutrophil extracellular traps
NKRF: NF-κB repressing factor
PD-1: programmed cell death 1
PD-L1: programmed cell death 1 ligand 1
RNA-seq: RNA sequencing
RT-qPCR: real-time quantitative PCR
SiglecF: sialic acid binding Ig-like lectin F
TANs: tumor-associated neutrophils
TCGA: the Cancer Genome Atlas
TDEs: tumor-derived exosomes
Tim3: T cell immunoglobulin and mucin domain-containing protein 3
TME: tumor microenvironment
WT: wild-type
