# Supplementary material for: Tumor-derived exosomes induce neutrophil infiltration and reprogramming to promote T-cell exhaustion in hepatocellular carcinoma
Source: Theranostics. 2025 Feb 3;15(7):2852–69. doi: 10.7150/thno.104557 (PMC11898284; doi:10.7150/thno.104557)

Tumor-derived exosomes induce neutrophil infiltration and reprogramming to promote T-cell exhaustion in hepatocellular carcinoma

Wenchao Bi[1], Xue Li[2], Yu Jiang[1], Tongtong Gao[1], Huajun Zhao[1], Qiuju Han[1], Jian Zhang[1]

[1]Institute of Immunopharmaceutical Sciences, School of Pharmaceutical Sciences, Shandong University, Jinan, China.

[2]Department of Medicinal Chemistry, Key Laboratory of Chemical Biology (Ministry of Education), School of Pharmaceutical Sciences, Shandong University, Jinan, China.

Correspondence Author: Qiuju Han, Institute of Immunopharmaceutical Sciences, School of Pharmaceutical Sciences, Shandong University, Jinan, 250012, China. Email: hanqiuju@sdu.edu.cn; or Jian Zhang, Institute of Immunopharmaceutical Sciences, School of Pharmaceutical Sciences, Shandong University, Jinan, 250012, China. Email: zhangj65@sdu.edu.cn.

Supplement Information

Supplementary Material and Methods

Supplementary Tables 1-4

Supplementary Figures 1-12

Raw imaging

Supplementary Material and Methods

Transmission electron microscopy

Exosomes were suspended in 1×PBS and dropped on formvar-carbon-coated grids for 1 min. Absorb excess liquid by filter paper and wash with deionized water. Then, the exosomes were negatively stained with 1% phosphotungstic acid. After drying, the morphology of exosomes was characterized by a JEM-1011 transmission electron microscope (JEOL, Japan).

Nanoparticle size analysis

Exosomes were diluted with 1×PBS. The particle size distribution of exosomes was analyzed by Malvern Zetasizer Nano ZS-90 (Malvern, UK).

Transwell assay

Tranwell plate (Corning, 3415) was used to evaluate neutrophil migration. Briefly, neutrophils (1×10^6) treated with or without SB225002 were suspended in 100 μL RPMI 1640 medium and added to the upper chamber. HCC exosomes suspended in 500 μL RPMI 1640 medium or conditioned medium of indicated neutrophils were added to the lower chamber. After 12 h, cells in the lower chambers were counted to determine the migrated neutrophil number.

Cell apoptosis assay

Neutrophil apoptosis was analyzed by using an Annexin V/APC apoptosis detection kit (62700-80, Biogems) or Annexin V-FITC/PI apoptosis detection kit (40302, Yeasen) according to the manufacturer’s instructions. For liver-infiltrating

neutrophils of mice, liver mononuclear cells were blocked with rat serum and then stained with anti-CD11b and anti-Ly6G fluorochrome-conjugated antibodies, followed by apoptosis detection. Data were collected using the BD FACSCelesta system.

Co-culture of neutrophils and T cells

The mouse spleen was passed through a 200-μm nylon cell strainer to obtain a single-cell suspension. Primary T cells were sorted using the EasySep™ Mouse T Cell Isolation Kit (19851, Stemcell). Splenic T cells (1×10^6) were cultured in a 12-well plate with RPMI 1640 medium supplemented with 10% FBS, 100 U/mL penicillin, and 100 mg/mL streptomycin. Anti-mouse CD3ε antibody (1 μg/mL, 1100340, Biolegend) and anti-mouse CD28 antibody (4 μg/mL, 102116, Biolegend) were added. At the same time, mBMDNs (1×10^6) pre-treated with or without Hepa 1-6-exosomes were added. After 48 h, these cells were collected, and T cell function was analyzed by flow cytometry.

Hematoxylin-eosin staining

Hematoxylin-eosin staining experiment was completed with the assistance of Lilai Biotechnology Co., Ltd. (Chengdu, China). Briefly, liver tissues were fixed with 4% paraformaldehyde, followed by dehydration, transparency, and paraffin-embedding. Paraffin sections were dewaxed to water and sequentially stained with hematoxylin and eosin. Dehydrate and transparent the slices again, and finally sealed with neutral gum. Images were captured using VS120 microscope (Olympus, Japan).

Immunofluorescence and confocal microscopy

To detect the uptake of exosomes by neutrophils, exosomes were stained with PKH26 (UR52302, Umibio) and added to neutrophils for 4 h. Then, the cells were fixed with 4% paraformaldehyde at 37 °C for 15 min and nuclei were stained with the Antifade Mounting Medium with DAPI (P0131, Beyotime). Images were acquired and analyzed under a Zeiss LSM 900 with Airyscan 2 software (Carl Zeiss, Germany).

Bioinformatics analysis

Infiltration of neutrophils in liver cancer or normal liver was determined by GEPIA2021 (gepia2021.cancer-pku.cn) analysis of TCGA and GTEx databases [1]. Infiltration of neutrophils in different stages of liver cancer was determined by the Assistant for Clinical Bioinformatics (www.aclbi.com) analysis of TCGA databases. Overall survival of liver cancer patients with high or low levels of neutrophil infiltration scores was determined by TIMER2.0 (timer.cistrome.org) analysis of TCGA databases [2]. Expression of miRNA in liver cancer and circulation was determined by CancerMIRNome (bioinfo.jialab-ucr.org/CancerMIRNome/) analysis of TCGA databases [3]. Expression of CYLD and NKRF in liver cancer or normal liver was determined by GEPIA2 (gepia2.cancer-pku.cn) analysis of TCGA databases [4]. Protein expression of CYLD and NKRF in liver cancer or normal liver was determined by UALCAN (ualcan.path.uab.edu) analysis of data from the Clinical Proteomic Tumor Analysis Consortium (CPTAC) [5]. The correlation of miR-362-5p expression and prognosis in liver cancer patients was determined by Kaplan-Meier Plotter (www.kmplot.com/analysis/) [6].

References

1. Li C, Tang Z, Zhang W, Ye Z, Liu F. GEPIA2021: integrating multiple deconvolution-based analysis into GEPIA. Nucleic Acids Res. 2021; 49: W242-W6.

2. Li T, Fu J, Zeng Z, Cohen D, Li J, Chen Q, et al. TIMER2.0 for analysis of tumor-infiltrating immune cells. Nucleic Acids Res. 2020; 48: W509-W14.

3. Li R, Qu H, Wang S, Chater JM, Wang X, Cui Y, et al. CancerMIRNome: an interactive analysis and visualization database for miRNome profiles of human cancer. Nucleic Acids Res. 2022; 50: D1139-D46.

4. Tang Z, Kang B, Li C, Chen T, Zhang Z. GEPIA2: an enhanced web server for large-scale expression profiling and interactive analysis. Nucleic Acids Res. 2019; 47: W556-W60.

5. Chandrashekar DS, Bashel B, Balasubramanya SAH, Creighton CJ, Ponce-Rodriguez I, Chakravarthi BVSK, et al. UALCAN: A Portal for Facilitating Tumor Subgroup Gene Expression and Survival Analyses. Neoplasia. 2017; 19: 649-58.

6. Győrffy B. Integrated analysis of public datasets for the discovery and validation of survival-associated genes in solid tumors. Innovation (Camb). 2024; 5: 100625.

Supplementary Tables

Table S1. Antibodies used in Western blotting and their technical information.

Name	Supplier	Cat No.	Clone No.
Anti-CD9	Abcam	ab92726	EPR2949
Anti-β-actin	Abclonal	AC026	ARC5115-01
Anti-CYLD	Abclonal	A3821	N/A
Anti-GRP94	Abclonal	A0989	N/A
Anti-TSG101	Abclonal	A22166	N/A
Anti-GAPDH	Abways	AB0036	N/A
Anti-NF-kB p65	Affinity	AF5006	N/A
Anti-Phospho-NF-kB p65 (Ser536)	Affinity	AF2006	N/A
Anti-Alix	CST	92880S	E6P9B
Anti-CD81	CST	10037S	D5O2Q
Anti-Histone H3	CST	4499S	D1H2
Anti-CD81	Santa Cruz	sc-166029	B-11
Anti-CD9	Santa Cruz	sc-13118	C-4

Table S2. Antibodies used in flow cytometry and their technical information.

Fluorescein	Antibody	Species	Cat No.	Supplier
AF700	CD45.2	Mus musculus	109822	Biolegend
APC	PD-1	Mus musculus	135210	Biolegend
APC	IFN-γ	Mus musculus	17-7311-82	eBioscience
APC	CD11b	Homo sapiens	301310	Biolegend
APC/Cyanine7	CD14	Mus musculus	123318	Biolegend
APC/Cyanine7	NK1.1	Mus musculus	108724	Biolegend
BUV737	Ly6G	Mus musculus	367-9668-82	eBioscience
BV421	IFN-γ	Mus musculus	505830	Biolegend
BV510	Fixable Viability Dye	Mus musculus	65-0866-14	eBioscience
BV605	PD-L1	Mus musculus	124321	Biolegend
BV711	IFN-γ	Mus musculus	564336	BD Horizon
FITC	CD11b	Mus musculus	11-0112-85	eBioscience
FITC	CD15	Homo sapiens	11-0159-42	eBioscience
FITC	CD3	Mus musculus	100204	Biolegend
PE	CD11b	Mus musculus	12-0112-83	eBioscience
PE	TNF-α	Mus musculus	12-7321-82	eBioscience
PE	CD69	Mus musculus	12-0691-83	eBioscience
PE	CD8a	Mus musculus	12-0081-83	eBioscience
PE-CF594	CD8a	Mus musculus	100762	Biolegend
PE-Cy7	TIGIT	Mus musculus	142108	Biolegend
PE-Cy7	TNF-α	Mus musculus	25-7321-82	eBioscience
PerCP/Cyanine5.5	CD3	Mus musculus	100218	Biolegend
PerCP/Cyanine5.5	Ly6G	Mus musculus	127616	Biolegend
PerCP/Cyanine5.5	Tim3	Mus musculus	119718	Biolegend

Table S3. Primer sequences for RT-qPCR.

**Gene**	**Species**	**Primer (5'-3')**	
β-actin	Homo sapiens	Forward	CACCATTGGCAATGAGCGGTTC
		Reverse	AGGTCTTTGCGGATGTCCACGT
CXCL1	Homo sapiens	Forward	AGCTTGCCTCAATCCTGCATCC
		Reverse	TCCTTCAGGAACAGCCACCAGT
CXCL2	Homo sapiens	Forward	GGCAGAAAGCTTGTCTCAACCC
		Reverse	CTCCTTCAGGAACAGCCACCAA
CXCL8	Homo sapiens	Forward	GAGAGTGATTGAGAGTGGACCAC
		Reverse	CACAACCCTCTGCACCCAGTTT
IL-1β	Homo sapiens	Forward	CCACAGACCTTCCAGGAGAATG
		Reverse	GTGCAGTTCAGTGATCGTACAGG
IL12b	Homo sapiens	Forward	GACATTCTGCGTTCAGGTCCAG
		Reverse	CATTTTTGCGGCAGATGACCGTG
TNF-α	Homo sapiens	Forward	CTCTTCTGCCTGCTGCACTTTG
		Reverse	ATGGGCTACAGGCTTGTCACTC
TGF-β	Homo sapiens	Forward	TACCTGAACCCGTGTTGCTCTC
		Reverse	GTTGCTGAGGTATCGCCAGGAA
MMP9	Homo sapiens	Forward	GCCACTACTGTGCCTTTGAGTC
		Reverse	CCCTCAGAGAATCGCCAGTACT
Bv8	Homo sapiens	Forward	GCTGCCATCCACTGACTCGTAA
		Reverse	CTCCAGAGCGATTACTTTTGGGC
CCL2	Homo sapiens	Forward	AGAATCACCAGCAGCAAGTGTCC
		Reverse	TCCTGAACCCACTTCTGCTTGG
p50	Homo sapiens	Forward	GCAGCACTACTTCTTGACCACC
		Reverse	TCTGCTCCTGAGCATTGACGTC
p52	Homo sapiens	Forward	GGCAGACCAGTGTCATTGAGCA
		Reverse	CAGCAGAAAGCTCACCACACTC
p65	Homo sapiens	Forward	TGAACCGAAACTCTGGCAGCTG
		Reverse	CATCAGCTTGCGAAAAGGAGCC
BIRC3	Homo sapiens	Forward	GCTTTTGCTGTGATGGTGGACTC
		Reverse	CTTGACGGATGAACTCCTGTCC
TRAF1	Homo sapiens	Forward	CGATGGCACTTTCCTGTGGAAG
		Reverse	TACAGCCGCAGGCACAACTTGT

β-actin	Mus musculus	Forward	CATTGCTGACAGGATGCAGAAGG
		Reverse	TGCTGGAAGGTGGACAGTGAGG
CXCL1	Mus musculus	Forward	TCCAGAGCTTGAAGGTGTTGCC
		Reverse	AACCAAGGGAGCTTCAGGGTCA
CXCL2	Mus musculus	Forward	CATCCAGAGCTTGAGTGTGACG
		Reverse	GGCTTCAGGGTCAAGGCAAACT
IL-1β	Mus musculus	Forward	TGGACCTTCCAGGATGAGGACA
		Reverse	GTTCATCTCGGAGCCTGTAGTG
IL12b	Mus musculus	Forward	TTGAACTGGCGTTGGAAGCACG
		Reverse	CCACCTGTGAGTTCTTCAAAGGC
TNF-α	Mus musculus	Forward	GGTGCCTATGTCTCAGCCTCTT
		Reverse	GCCATAGAACTGATGAGAGGGAG
TGF-β	Mus musculus	Forward	TGATACGCCTGAGTGGCTGTCT
		Reverse	CACAAGAGCAGTGAGCGCTGAA
Bv8	Mus musculus	Forward	TGCTGTGCTGTCAGTATCTGGG
		Reverse	TTCGCCCTTCTTCTTTCCTGCC
CCL2	Mus musculus	Forward	GCTACAAGAGGATCACCAGCAG
		Reverse	GTCTGGACCCATTCCTTCTTGG
RNU6	Homo sapiens<br>Mus musculus	Forward<br>Reverse	CTCGCTTCGGCAGCACA<br>AACGCTTCACGAATTTGCGT
miR-196b-5p	Homo sapiens<br>Mus musculus	Forward	CCCTAGGTAGTTTCCTGTTGTTGGG
miR-205-5p	Homo sapiens<br>Mus musculus	Forward	TCCTTCATTCCACCGGAGTCTG
miR-342-3p	Homo sapiens<br>Mus musculus	Forward	TCTCACACAGAAATCGCACCCG
miR-21-5p	Homo sapiens<br>Mus musculus	Forward	GGCTAGCTTATCAGACTGATGTTGA
miR-301a-3p	Homo sapiens<br>Mus musculus	Forward	GGCAGTGCAATAGTATTGTCAAAGC
miR-362-5p	Homo sapiens<br>Mus musculus	Forward	CAATCCTTGGAACCTAGGTGTGAGT

Table S4. Primer sequences for plasmid construction.

**Plasmid**	**Primer (5'-3')**	
WT-CYLD-1	Forward	CTAGCTAGCAAGTATGTTTGTGTTGGTTT
	Reverse	CGGCTAGCCAACACCATTAAGGGAATTT
WT-CYLD-2	Forward	CTAGCTAGCGAGACTAGGGTTTTAGACTG
	Reverse	CGGCTAGCTCCCTCCATTTGTGGCCCTT
Mut-CYLD-1	Forward	ATGGACGTTCCTAATGAAATCATTTTTCTTTTGTAGCTA
	Reverse	TTTCATTAGGAACGTCCATAAAGAAGACACATTTATTTA
Mut-CYLD-2	Forward	TGTGGCGTTCCTAAATGAAAAAGTGGGAAAGGAAGGTCC
	Reverse	TTCATTTAGGAACGCCACACTTGCTGGTTAATGCATTAC

Supplementary Figures 1-12

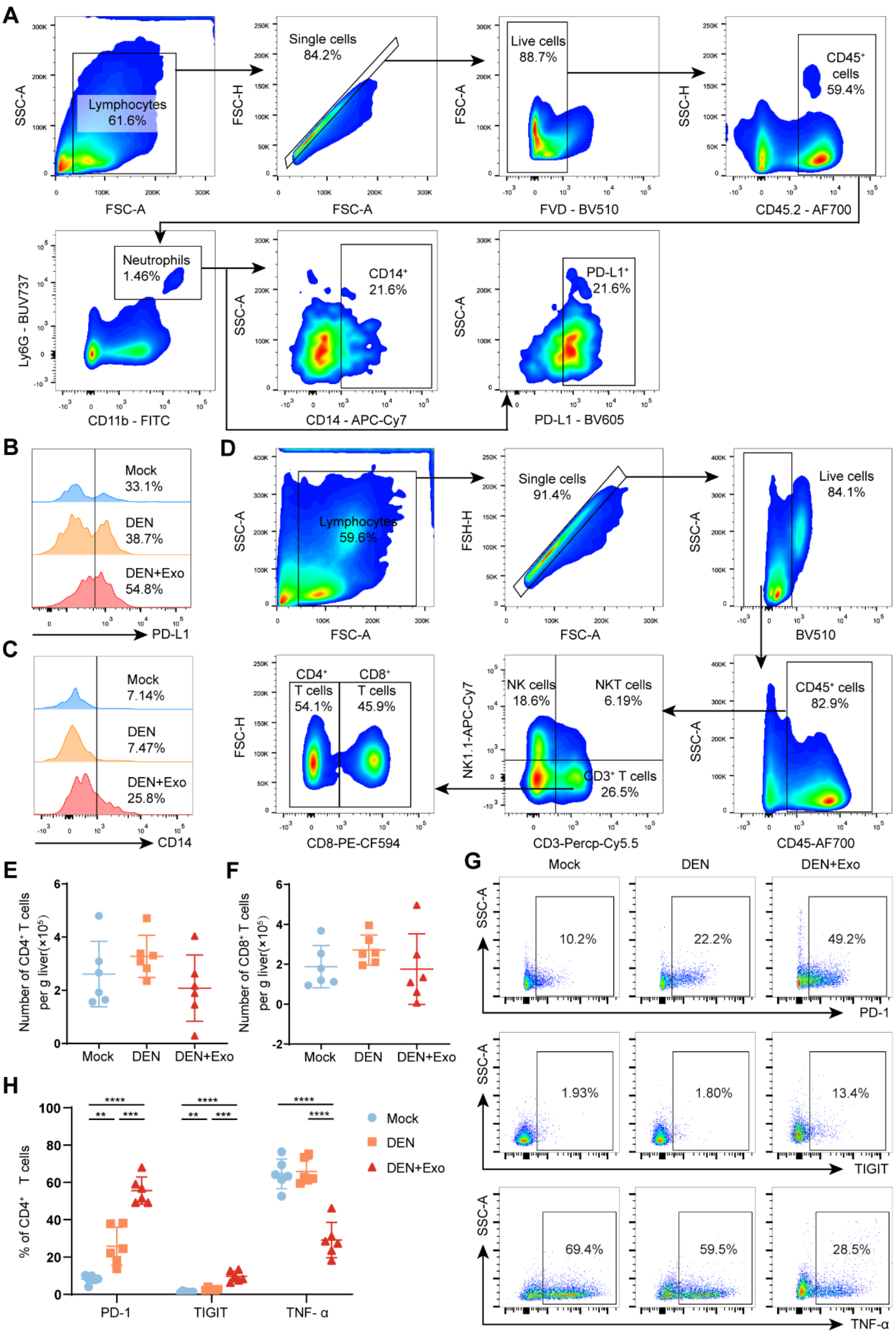

Figure S1. HCC exosome induced $CD4^+$ T-cell exhaustion in the liver of HCC mice.

(**A**) The gating strategy for liver-infiltrating neutrophils. (**B, C**) The proportions of PD-$L1^+$ neutrophils and $CD14^+$ neutrophils in the liver were analyzed by flow cytometry. (**D**) The gating strategy for liver-infiltrating T cells. (**E-H**) Flow cytometry was performed to analyze the number of $CD4^+$ T cells (**E**) and $CD8^+$ T cells (**F**) in the liver, and the expression of PD-1, TIGIT, and TNF-α on liver-infiltrating $CD4^+$ T cells (**G, H**). Mock, healthy mice; DEN, DEN/CCl_4-induced HCC mice; Exo, exosomes. Data are presented as mean ± S.D. from at least three independent experiments. *$p < 0.05$, **$p < 0.01$, ***$p < 0.001$, and ****$p < 0.0001$.

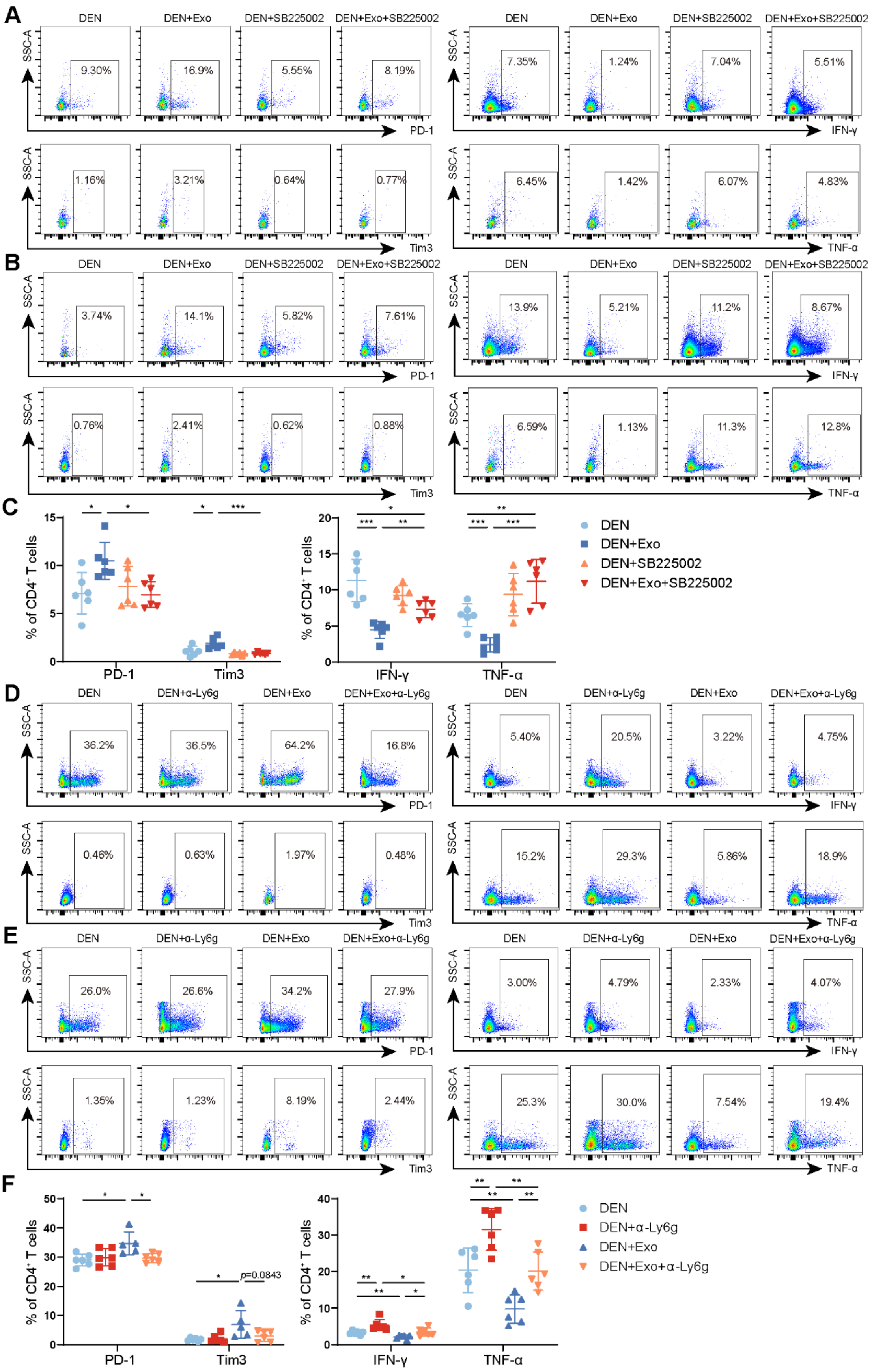
A
DEN
DEN+Exo
DEN+SB225002
DEN+Exo+SB225002
SSC-A
9.30%
16.9%
5.55%
8.19%
PD-1
7.35%
1.24%
7.04%
5.51%
IFN-γ
1.16%
3.21%
0.64%
0.77%
Tim3
6.45%
1.42%
6.07%
4.83%
TNF-α
B
3.74%
14.1%
5.82%
7.61%
13.9%
5.21%
11.2%
8.67%
0.76%
2.41%
0.62%
0.88%
6.59%
1.13%
11.3%
12.8%
C
% of CD4+ T cells
PD-1
Tim3
IFN-γ
TNF-α
DEN
DEN+Exo
DEN+SB225002
DEN+Exo+SB225002
D
DEN
DEN+α-Ly6g
DEN+Exo
DEN+Exo+α-Ly6g
36.2%
36.5%
64.2%
16.8%
5.40%
20.5%
3.22%
4.75%
0.46%
0.63%
1.97%
0.48%
15.2%
29.3%
5.86%
18.9%
E
26.0%
26.6%
34.2%
27.9%
3.00%
4.79%
2.33%
4.07%
1.35%
1.23%
8.19%
2.44%
25.3%
30.0%
7.54%
19.4%
F
p=0.0843
DEN
DEN+α-Ly6g
DEN+Exo
DEN+Exo+α-Ly6g

Figure S2. Blocking the hepatic infiltration of neutrophils alleviates HCC exosome-induced T-cell exhaustion.

(**A-F**) Flow cytometry was performed to analyze the expression of PD-1, Tim3, IFN-γ, and TNF-α on liver-infiltrating $CD8^+$ T cells (**A, D**) and $CD4^+$ T cells (**B, C, E, and F**) in mice. DEN, DEN/CCl_4-induced HCC mice; Exo, exosomes; α-Ly6g, anti-Ly6g blocking antibody. Data are presented as mean ± S.D. from at least three independent experiments. *$p < 0.05$, **$p < 0.01$, and ***$p < 0.001$.

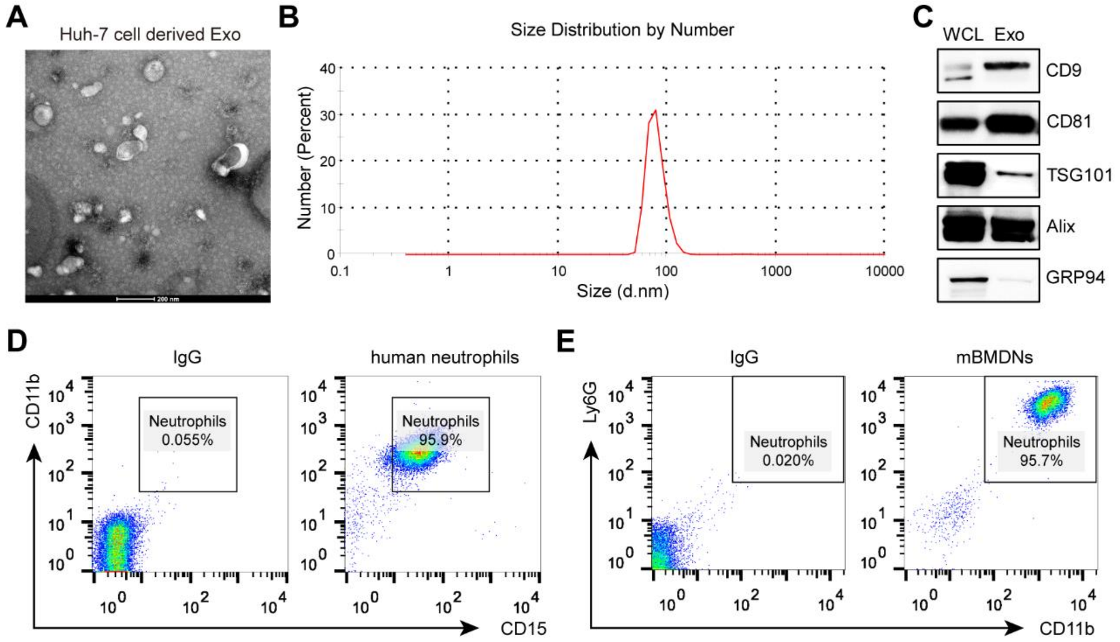

Figure S3. The identification of Huh-7 cell-exosomes and the purity of isolated neutrophils.

(**A–C**) Huh-7 cell-exosomes were isolated by ultracentrifugation and identified by transmission electron microscopy (**A**), particle size analyzer (**B**), and western blotting (**C**). (**D, E**) The purity of neutrophils isolated from human peripheral blood (**D**) or mouse bone marrow (**E**) was determined by flow cytometry. WCL, whole cell lysate; Exo, exosomes; mBMDNs, mouse bone marrow-derived neutrophils. One representative of at least three independent experiments.

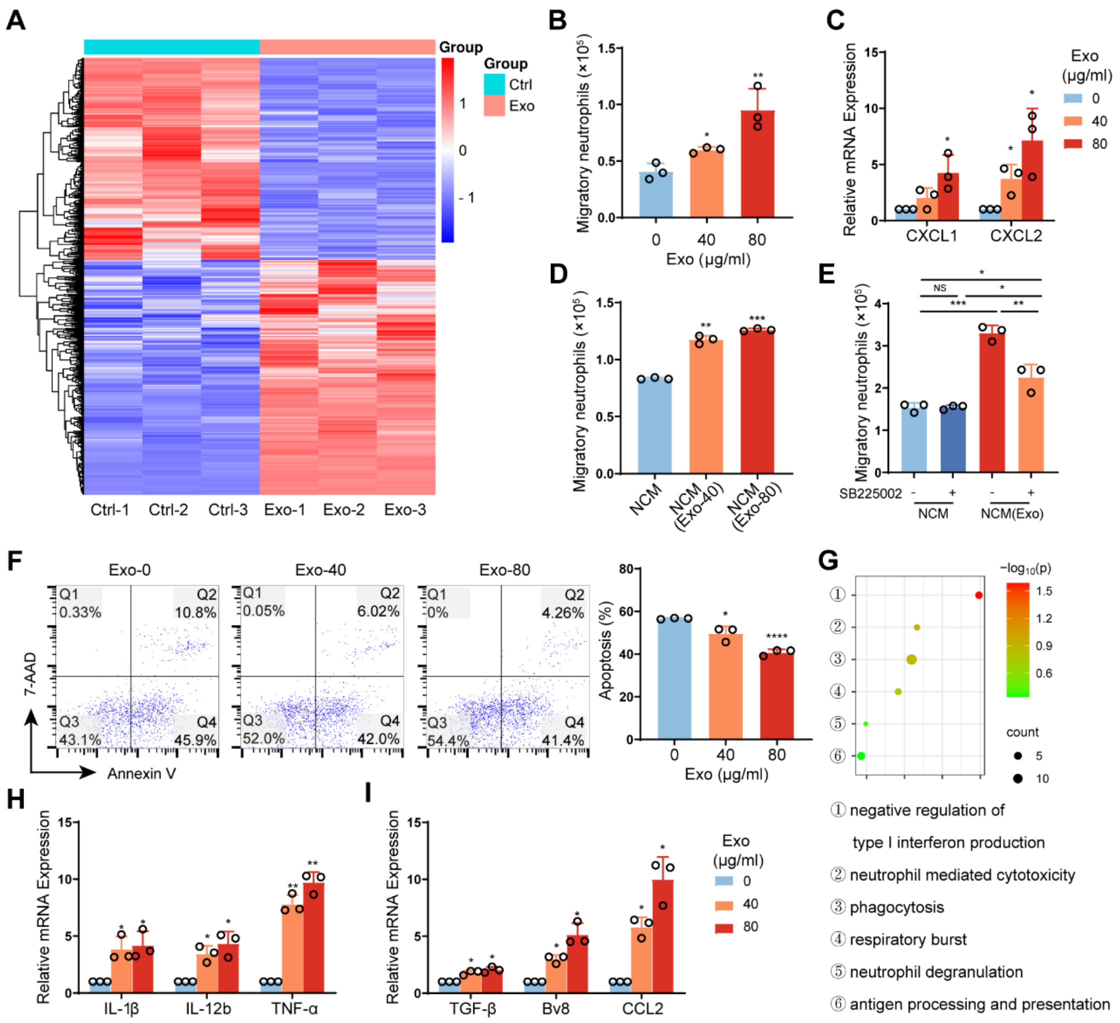

Figure S4. HCC-exosomes induced migration, survival, and protumor polarization of mBMDNs *in vitro*.

(**A**) Heatmap of differentially expressed genes in neutrophils treated with or without Huh-7 cell-exosomes. (**B**) Hepa 1-6 cell-exosomes were added to recruit mBMDNs. After 12 h, the migration ability of mBMDNs was determined by the number of mBMDNs in the lower chamber. (**C**) RT–qPCR assay was performed to detect the expression of chemokines (CXCLs) in mBMDNs treated with or without Hepa 1-6 cell-exosomes. (**D, E**) NCM and NCM(Exo) were added to recruit mBMDNs treated

with or without SB225002 (400nM). After 12 h, the migration ability of mBMDNs was determined by the number of mBMDNs in the lower chamber. (**F**) The apoptosis of mBMDNs treated with or without Hepa 1-6 cell-exosomes for 12 h was detected by Annexin V/7AAD staining. (**G**) GO enrichment analysis of differentially expressed genes related to N1-polarization pathways in human neutrophils treated with or without Huh-7 cell-exosomes. (**H, I**) RT–qPCR was performed to detect the expression of inflammation (**H**) and polarization (**I**) related genes in mBMDNs treated with or without Hepa 1-6 cell-exosomes. Exo, exosomes; NCM, conditioned medium from mBMDNs; NCM(Exo), conditioned medium from mBMDNs treated with Hepa 1-6 cell-exosomes. Data are presented as mean ± S.D. from at least three independent experiments. *$p < 0.05$, **$p < 0.01$, ***$p < 0.001$, and ****$p < 0.0001$.

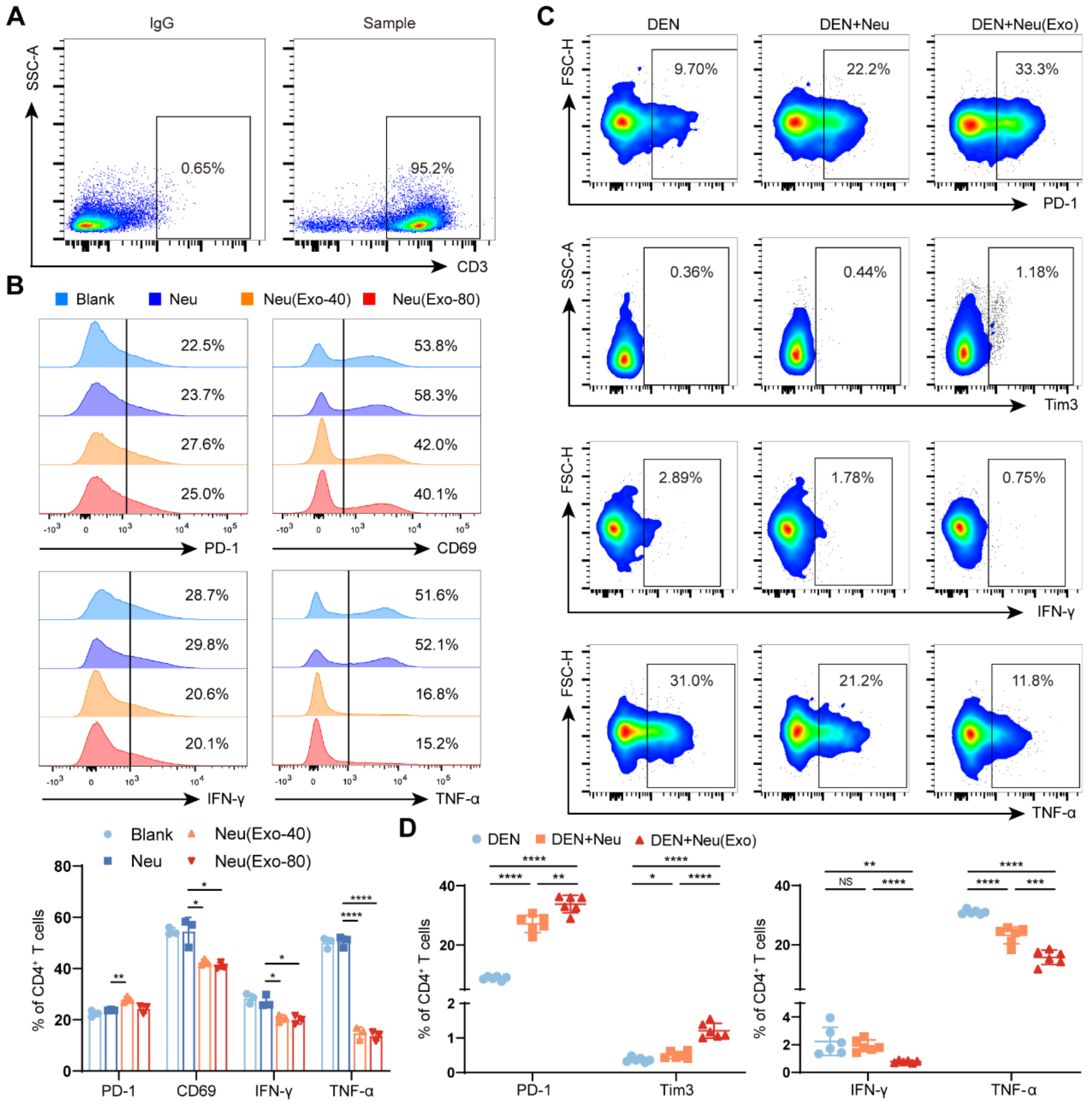

Figure S5. HCC exosome-reprogrammed neutrophils induce CD4$^+$ T cell exhaustion.

(**A**) The purity of splenic T cells was determined by flow cytometry. (**B**) Splenic T cells isolated from healthy mice were co-cultured with mBMDNs treated with or without Hepa 1-6 cell-exosomes for 48 h. Flow cytometry was performed to analyze the expression of PD-1, CD69, IFN-γ, and TNF-α on CD4$^+$ T cells. (**C, D**) Flow cytometry was performed to analyze the expression of PD-1, Tim3, IFN-γ, and TNF-α

on liver-infiltrating $CD4^+$ T cells. DEN, DEN/CCl_4-induced HCC mice; Neu, mBMDNs; Neu(Exo), mBMDNs treated with Hepa 1-6 cell-exosomes; Exo, exosomes. Data are presented as mean ± S.D. from at least three independent experiments. *$p < 0.05$, **$p < 0.01$, ***$p < 0.001$, and ****$p < 0.0001$.

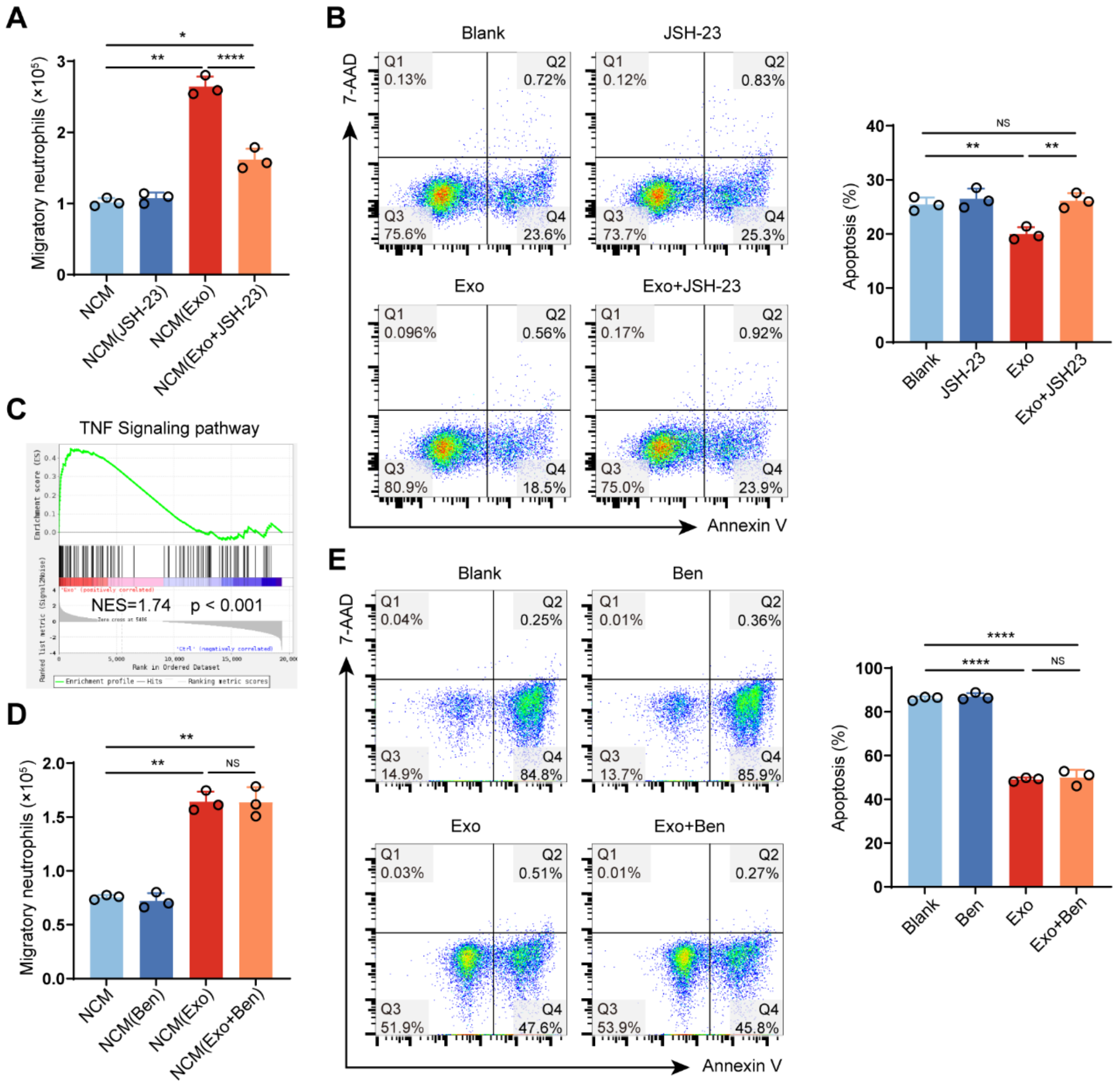

Figure S6. HCC exosomes reprogram mBMDNs by activating the NF-κB signaling pathway.

(**A, B**) mBMDNs were treated with JSH-23 (400 nM), Hepa 1-6 cell-exosomes (40 μg/mL), or the combination. Exosomes were added 2 h post JSH-23 treatment. The conditioned mediums from these mBMDNs were added to recruit mBMDNs (**A**). The apoptosis of mBMDNs was detected by Annexin V/7-AAD staining (**B**). (**C**) GSEA plot showing the enrichment scores for the TNF signaling pathway in human

neutrophils treated with Huh-7 cell-exosomes (40 μg/mL). (**D, E**) Human neutrophils were treated with Benpyrine (400 nM), Huh-7 cell-exosomes (40 μg/mL), or the combination. Exosomes were added 2 h post of Benpyrine treatment. The conditioned mediums from these neutrophils were added to recruit neutrophils (**D**). The apoptosis of neutrophils was detected by Annexin V/7-AAD staining (**E**). Exo, exosomes; NCM, conditioned medium from neutrophils; Ben, Benpyrine. Data are presented as mean ± S.D. from at least three independent experiments. *$p < 0.05$, **$p < 0.01$, ***$p < 0.001$, and ****$p < 0.0001$.

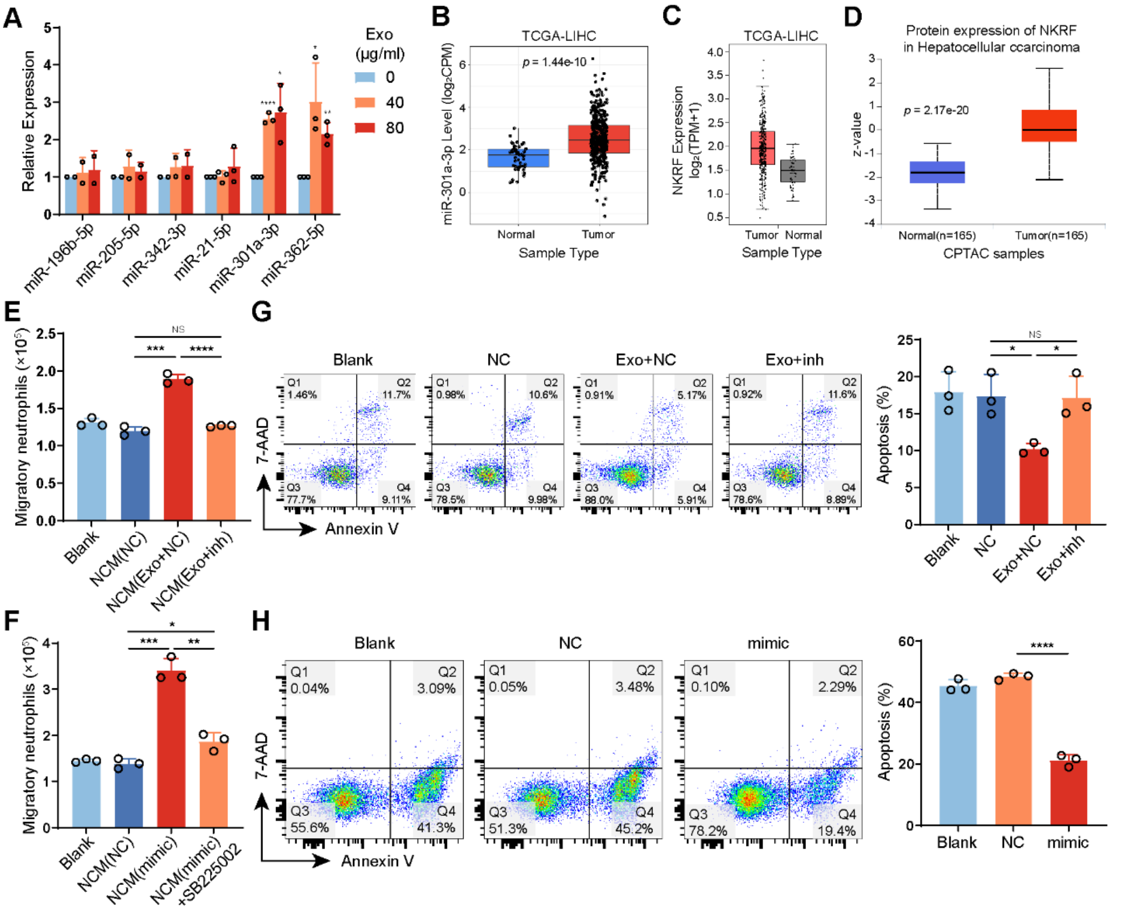

Figure S7. HCC exosomes induce miR-362-5p enrichment to promote chemotaxis and survival of mBMDNs.

(**A**) RT–qPCR assay was performed to detect the expression of miRNAs in mBMDNs treated with or without Hepa 1-6 cell-exosomes. (**B**) The levels of has-miR-301a-3p in liver cancer or normal liver were evaluated by using the CancerMIRNome database. (**C**) The levels of NKRF mRNA in liver cancer or normal liver were evaluated by using the GEPIA2 database. (**D**) The protein levels of NKRF in liver cancer or normal liver were evaluated by using the UALCAN database. (**E, G**) mBMDNs were transfected with NC or miR-362-5p inhibitor for 4 h and then treated with Hepa 1-6 cell-exosomes (40 μg/mL) for another 12 h. The conditioned mediums as indicated

were collected to recruit mBMDNs (**E**). The apoptosis of mBMDNs was detected by Annexin V/7-AAD staining (**G**). (**F, H**) mBMDNs were transfected with NC or miR-362-5p mimic for 12 h. The conditioned mediums as indicated were collected to recruit mBMDNs treated with or without SB225002 (400nM) (**F**). The apoptosis of mBMDNs was detected by Annexin V-7-AAD staining (**H**). NC, negative control; inh, miR-362-5p inhibitor; Exo, exosomes; NCM, conditioned medium from neutrophils. Data are presented as mean ± S.D. from at least three independent experiments. *$p < 0.05$, **$p < 0.01$, ***$p < 0.001$, and ****$p < 0.0001$.

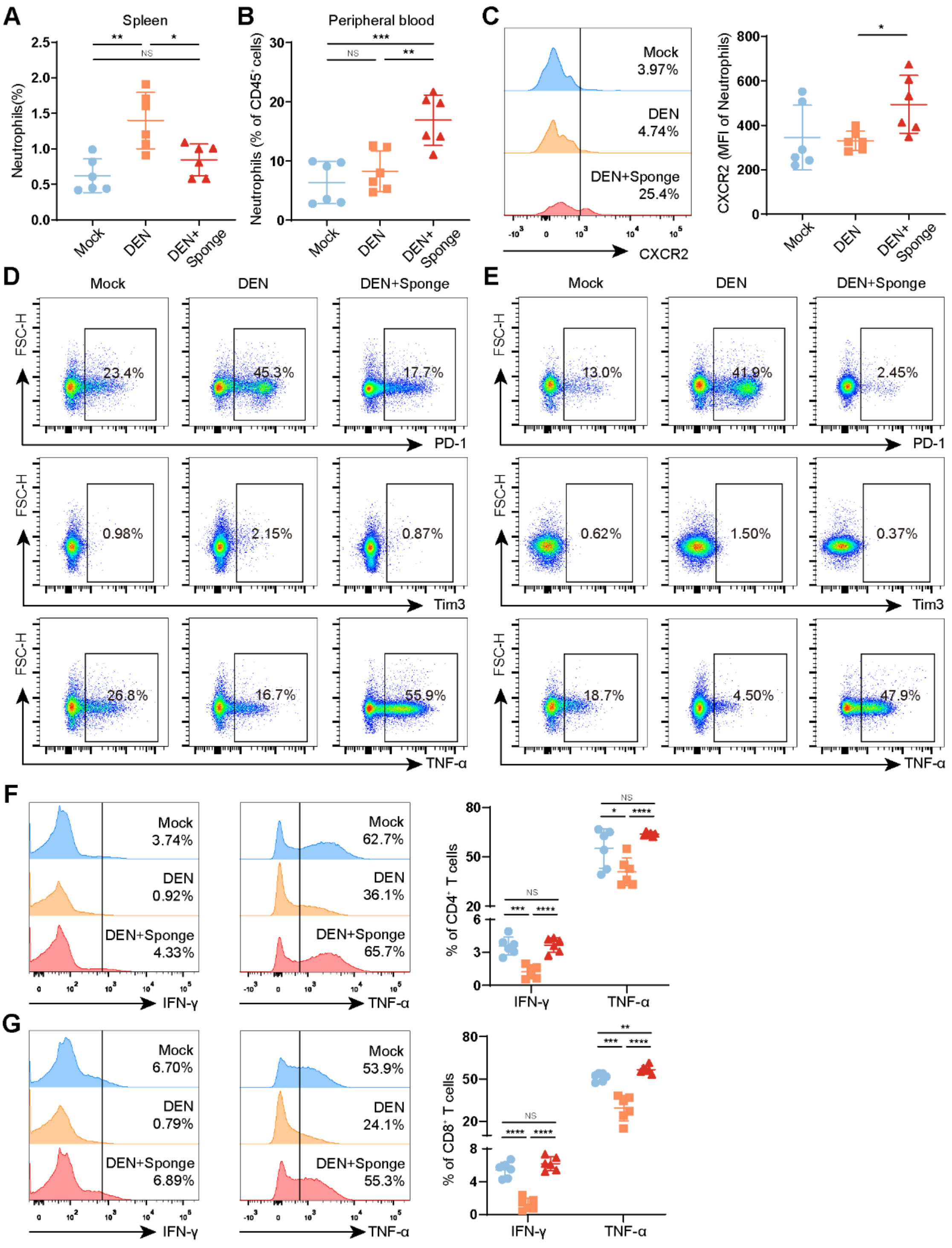

Figure S8. Blocking miR-362-5p promotes neutrophil infiltration and reverses T-cell exhaustion.

(**A, B**) Leukocytes were harvested from the spleen (**A**) or peripheral blood (**B**) of mice, and the percentage of neutrophils was determined by flow cytometry. (**C**) The expression of CXCR2 on neutrophils from the peripheral blood of mice was determined by using flow cytometry. (**D, E**) The expressions of PD-1, Tim3, and TNF-α on liver-infiltrating $CD4^+$ T cells (**D**) and $CD8^+$ T cells (**E**) of mice were determined by flow cytometry. (**F, G**) The expressions of IFN-γ and TNF-α on splenic $CD4^+$ T cells (**F**) and $CD8^+$ T cells (**G**) of mice were determined by flow cytometry. Mock, healthy mice; DEN, DEN/CCl_4-induced mice. Data are presented as mean ± S.D. from at least three independent experiments. *$p < 0.05$, **$p < 0.01$, ***$p < 0.001$, and ****$p < 0.0001$.

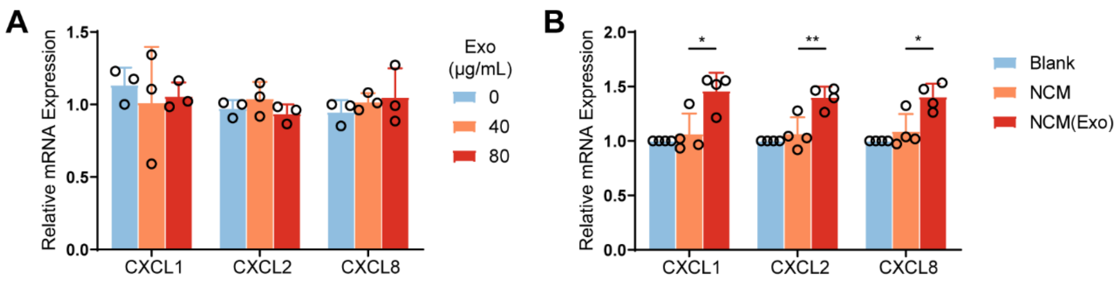

Figure S9. HCC exosome-reprogrammed neutrophils upregulate the expression of CXCL1, CXCL2, and CXCL8 in HCC cells.

(**A**) Huh-7 cells were treated with Huh-7 cell-exosomes. RT–qPCR assay was performed to detect the expression of chemokines (CXCLs) in Huh-7 cells. (**B**) Huh-7 cells were treated with conditioned medium from neutrophils and Huh-7 cell-exosome-reprogrammed neutrophils. RT–qPCR assay was performed to detect the expression of chemokines (CXCLs) in Huh-7 cells. Exo, exosomes; NCM, conditioned medium from human neutrophils; NCM(Exo), conditioned medium from human neutrophils treated with Huh-7 cell-exosomes. Data are presented as mean ± S.D. from at least three independent experiments. *$p < 0.05$ and **$p < 0.01$.

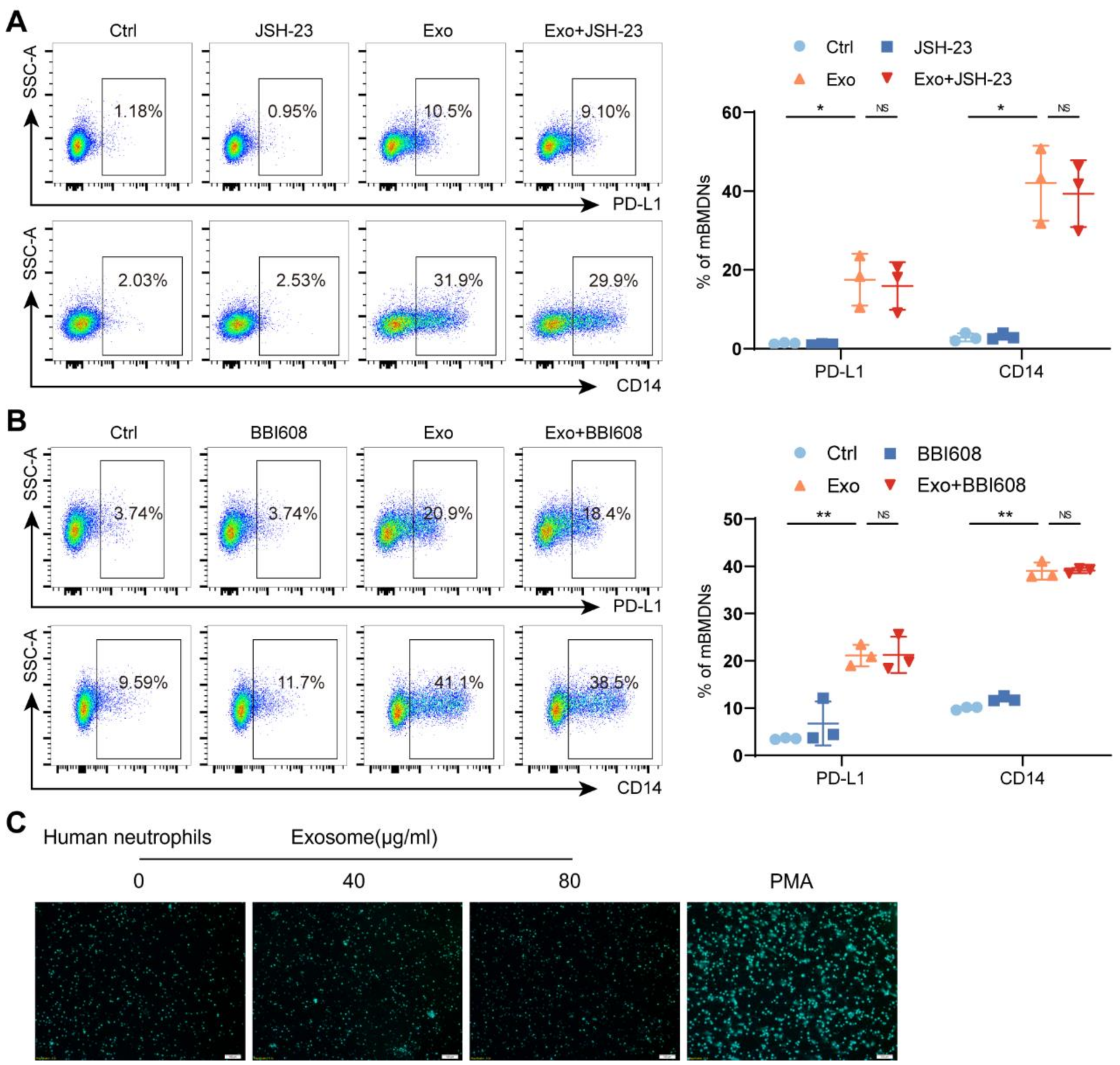

Figure S10. The mechanism of HCC exosome-induced immunosuppressive phenotype of neutrophils is unclear.

(**A**) mBMDNs were treated with JSH-23 (400 nM), Hepa 1-6 cell-exosomes (40 μg/mL), or the combination. Exosomes were added 2 h post JSH-23 treatment. The expressions of PD-L1 and CD14 on neutrophils were determined by flow cytometry. (**B**) mBMDNs were treated with BBI608 (100 nM), Hepa 1-6 cell-exosomes (40 μg/mL), or the combination. Exosomes were added 2 h post BBI608 treatment. The expressions of PD-L1 and CD14 on neutrophils were determined by flow cytometry.

(**C**) Human neutrophils treated with or without Huh-7 cell-exosomes or PMA (100 nM) for 12 h were stained with the SYTOX Green Nucleic Acid Stain to detect the formation of NETs. Exo, exosomes. Data are presented as mean ± S.D. from at least three independent experiments. *$p < 0.05$ and **$p < 0.01$.

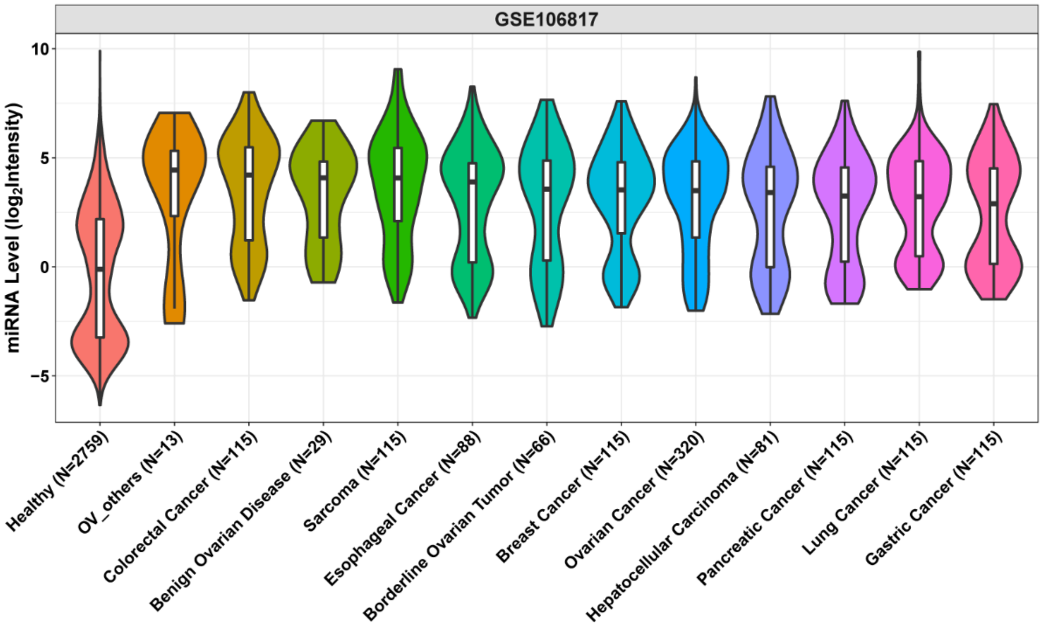

Figure S11. The expression of miR-362-5p in the circulation of cancer patients.

The expression of miR-362-5p in the circulation of cancer patients from the GSE106817 database.

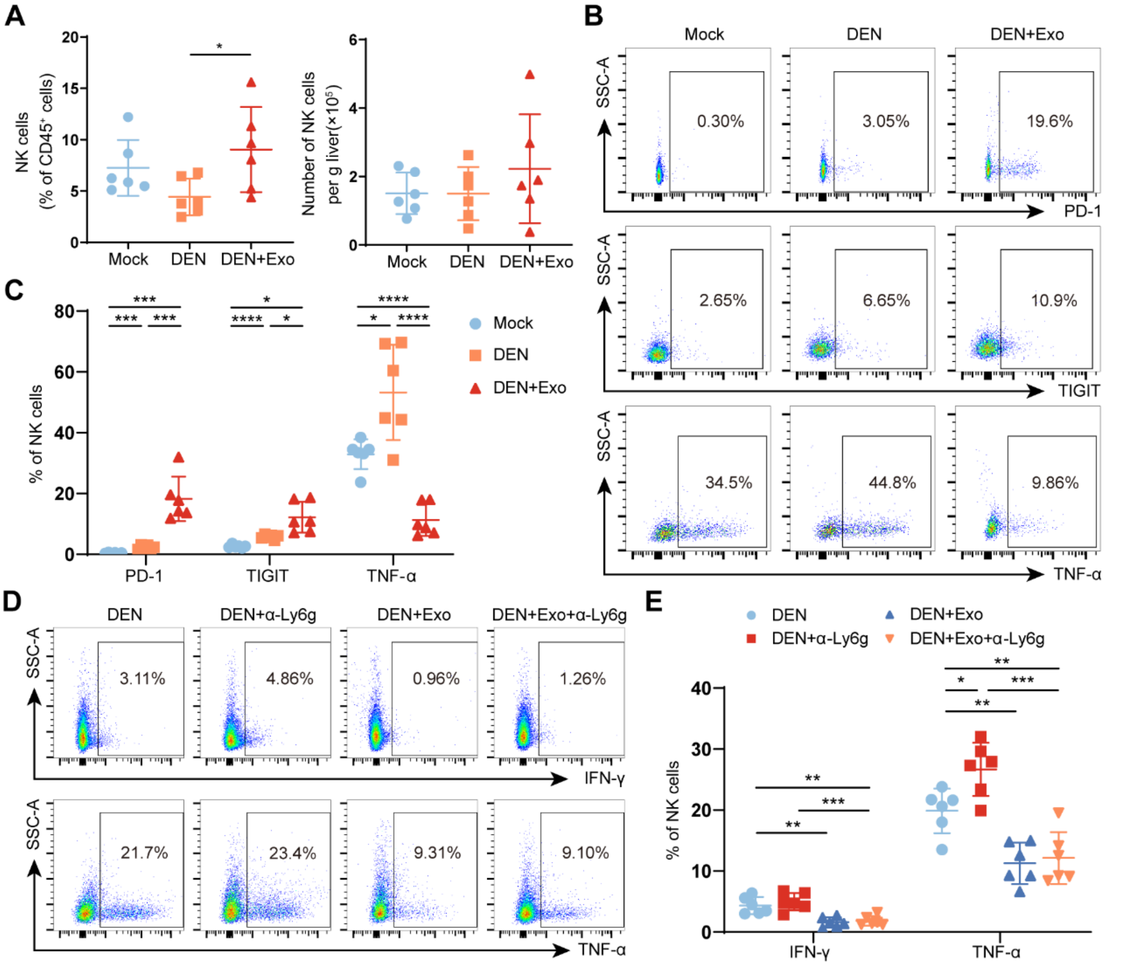

Figure S12. HCC exosome-induced exhaustion of NK cells is not dependent on neutrophils.

(**A-E**) Flow cytometry was performed to analyze the proportion and number of NK cells in the liver (**A**), and the expression of PD-1, TIGIT, TNF-α, and IFN-γ on liver-infiltrating NK cells (**B-E**). Mock, healthy mice; DEN, DEN/CCl_4-induced HCC mice; Exo, exosomes; α-Ly6g, anti-Ly6g blocking antibody. Data are presented as mean ± S.D. from at least three independent experiments. **p* < 0.05, ***p* < 0.01, ****p* < 0.001, and *****p* < 0.0001.

Raw imaging

Figure 1F

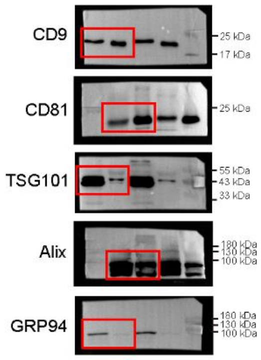

Figure 5D

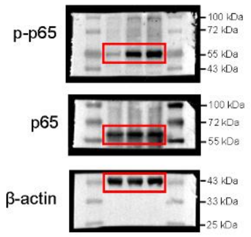

Figure 5E

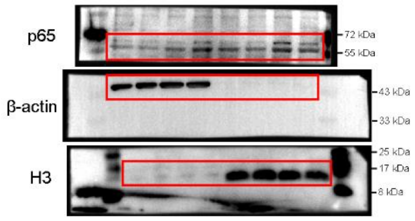

Figure 6M

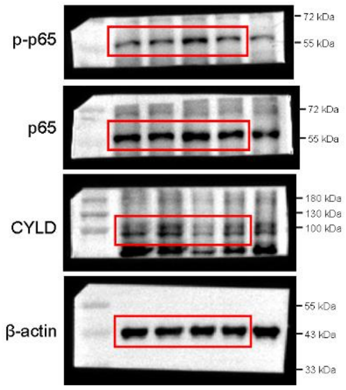

Figure 6N

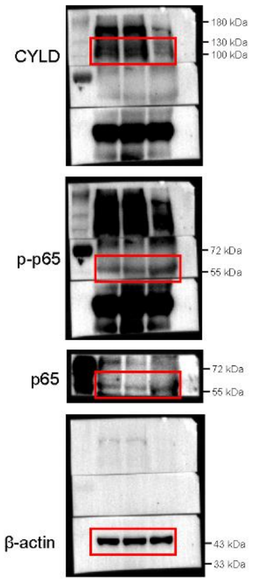

Figure 7D

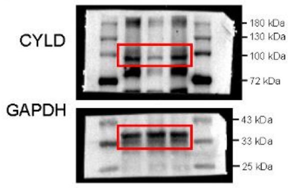

Figure S4C

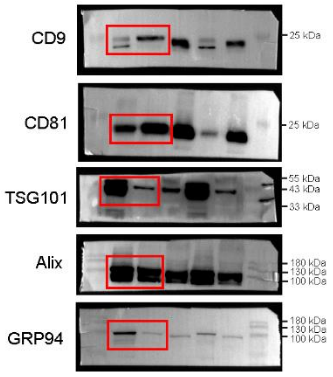

Supplement: Supplementary file 1 — Supplementary materials and methods, figures. [file thnov15p2852s1.pdf]
